# SUMF1 overexpression promotes tumorous cell growth and migration and is correlated with the immune status of patients with glioma

**DOI:** 10.18632/aging.205626

**Published:** 2024-03-07

**Authors:** Ping Zhang, Zhao Liu, Yu-Yu Wang, Hui-Jiu Luo, Chao-Zhi Yang, Hao Shen, Hai-Tao Wu, Ju-Hang Li, Hong-Xin Zhao, Qi-Shan Ran

**Affiliations:** 1 Department of Neurosurgery, Affiliated Hospital of Zunyi Medical University, Zunyi 563000, China; 2 Cancer Center, Union Hospital, Tongji Medical College, Huazhong University of Science and Technology, Wuhan 430022, China

**Keywords:** SUMF1, glioma, nomogram, risk models, biomarkers

## Abstract

Background: Glioma is a prevalent type of malignant tumor. To date, there is a lack of literature reports that have examined the association between sulfatase modifying factor 1 (SUMF1) and glioma.

Methods: The levels of SUMF1 were examined, and their relationships with the diagnosis, prognosis, and immune microenvironment of patients with glioma were investigated. Cox and Lasso regression analysis were employed to construct nomograms and risk models associated with SUMF1. The functions and mechanisms of SUMF1 were explored and verified using gene ontology, cell counting kit-8, wound healing, western blotting, and transwell experiments.

Results: SUMF1 expression tended to increase in glioma tissues. SUMF1 overexpression was linked to the diagnosis of cancer, survival events, isocitrate dehydrogenase status, age, and histological subtype and was positively correlated with poor prognosis in patients with glioma. SUMF1 overexpression was an independent risk factor for poor prognosis. SUMF1-related nomograms and high-risk scores could predict the outcome of patients with glioma. SUMF1 co-expressed genes were involved in cytokine, T-cell activation, and lymphocyte proliferation. Inhibiting the expression of SUMF1 could deter the proliferation, migration, and invasion of glioma cells through epithelial mesenchymal transition. SUMF1 overexpression was significantly associated with the stromal score, immune cells (such as macrophages, neutrophils, activated dendritic cells), estimate score, immune score, and the expression of the programmed cell death 1, cytotoxic T-lymphocyte associated protein 4, CD79A and other immune cell marker.

Conclusion: SUMF1 overexpression was found to be correlated with adverse prognosis, cancer detection, and immune status in patients with glioma. Inhibiting the expression of SUMF1 was observed to deter the proliferation, migration, and invasion of cancer cells. The nomograms and risk models associated with SUMF1 could predict the prognosis of patients with glioma.

## INTRODUCTION

Glioma is a prevalent type of primary malignant tumor found in the central nervous system. While substantial progress has been made in its treatment through surgery, radiotherapy, and chemotherapy, the recurrence and progression of glioma remain commonplace due to the tumor’s highly invasive properties. Furthermore, although extensive research has established the significant role played by the isocitrate dehydrogenase (IDH) mutation and the 1p/19q codeletion in the diagnosis, treatment, and prognostic assessment of glioma, the prognosis for afflicted patients remains unfavorable [1]. It is consequently imperative to explore novel approaches to the diagnosis and treatment of glioma.

Numerous studies have revealed correlations between changes in the expression of specific long non-coding RNAs/genes and the growth/metastasis of cancer [2–6]. For example, Ge et al., found an association between the overexpression of tumor protein p53 inducible protein 13 (TP53I13) in glioma tissues and unfavorable survival outcomes. TP53I13 overexpression is significantly correlated with IDH status, age, chemotherapy, 1p/19q codeletion, and tumor grade in glioma patients [3]. Furthermore, the expression of small G protein signaling modulator 1 (SGSM1) tended to be relatively diminished in low-grade glioma tissues. The downregulation of SGSM1 expression was found to be associated with poor survival time in patients with either low-grade glioma or its subtypes [5]. Numerous studies have confirmed a significant correlation between sulfatases (SULF) and cancer [7–12]. Specifically, Li et al., reported that sulfatase modifying factor 1 (SUMF1) was associated with the overall survival (OS) of patients with glioma. However, SUMF1 has yet to be implicated in any other aspect of glioma. This study aims to investigate the expression of SUMF1 and its associations with the prognosis, diagnosis, and immune microenvironment of patients with glioma. This investigation further explores the roles that SUMF1 plays in glioma progression, as well as the mechanisms underpinning its actions, by using gene ontology (GO) analysis, cell counting kit-8 (CCK-8), wound healing, western blotting, and Transwell experiments. The results of this research may lay new theoretical foundations for the treatment of glioma.

## MATERIALS AND METHODS

### Sources of information for patients with glioma

Transcript per million (TPM) data for five healthy brain tissues and 701 glioma tissues were obtained from the Cancer Genome Atlas (TCGA) database along with the clinical characteristics and information for 1121 patients with glioma. The XENA database includes transcriptome data from both the Genotype-Tissue Expression (GTEx) and TCGA databases. Consequently, we downloaded the TPM data for 1152 healthy brain tissues from the GTEx database. The data for the five healthy brain tissues and 689 glioma tissues was collected from the XENA database from TCGA database.

### SUMF1 expression in glioma tissues

After retrieving SUMF1 expression data from the transcriptome data available in the TCGA database, we determined the levels of SUMF1 in five healthy brain tissues and 701 glioma tissues through expression analysis. Additionally, we obtained SUMF1 expression data from the transcriptome data in the XENA database and assessed the expression levels of SUMF1 in 1157 healthy brain tissues and 689 glioma tissues through expression analysis.

### The correlation between SUMF1 expression and clinicopathological characteristics in patients with glioma

The transcriptome data of 701 glioma patients was merged with the clinical characteristics of 1121 patients with glioma using the Perl language. Data associated with incomplete clinical information were excluded. Patients were categorized into two groups based on their median values of SUMF1 expression. The relationship between changes in SUMF1 expression and characteristics such as IDH status, disease-specific survival (DSS) event, 1p/19q codeletion status, gender, age, histological subtype, OS event, and progression-free interval (PFI) event were determined for the patients with glioma. These data established the context for identifying the expression levels of SUMF1 in afflicted patients.

### Evaluating the diagnostic value of SUMF1 in glioma

Receiver operating characteristic (ROC) analysis is frequently employed to evaluate the diagnostic value of long non-coding RNAs or genes in cancer [13–15]. Transcriptome data from the TCGA and XENA databases were used to assess the diagnostic value of SUMF1 in glioma. Additionally, we performed the ROC analysis with data from the TCGA database to identify the relationship between the gene encoding SUMF1 and the prognosis of glioma patients over 1, 3, and 5 years. The evaluation criterion in ROC analysis was the area under the curve (AUC).

### Assessing the prognostic value of SUMF1 in glioma

The transcriptome data of 701 glioma patients were integrated with prognostic information from 1121 glioma patients using the Perl language. Data associated with incomplete clinical profiles were excluded. Patients with glioma were divided into two groups based on the median expression of SUMF1. The association between the changes in SUMF1 expression and glioma prognosis indicators (such as the survival time, PFI, and DSS) was determined using the Kaplan-Meier (K-M) survival analysis. A subgroup analysis of glioma patients was subsequently performed, and the relationship between the changes in SUMF1 and prognostic indicators (OS, PFI, and DSS) was determined using the K-M survival analysis. *P*-values of < 0.05 were considered indicative of statistical significance.

### COX regression analysis and construction of nomograms

We used information on SUMF1 expression, IDH status, 1p/19q encoding, gender, age, and histological subtypes to investigate the risk factors influencing OS, DSS, and PFI in patients with glioma. The results from the univariate COX regression analysis were filtered based on a significance index of *P* < 0.05. We subsequently identified independent risk factors that significantly impacted the prognosis of patients with glioma and constructed a nomogram related to SUMF1 with *P* < 0.05 as the bassline index of statistical significance.

### The biological function of genes co-expressed with SUMF1

Data collected from the TCGA was subjected to Spearman analysis to identify genes that were co-expressed with SUMF1 in glioma tissues. Genes exhibiting a correlation coefficient whose absolute value exceeded 0.6 were deemed to be strongly correlated with SUMF1. To understand the functions of these strongly co-expressed genes, we used GO annotations encompassing molecular function, cellular components, and biological processes [16, 17]. A significance level of *P* < 0.05 was adopted as the threshold for statistical significance.

### Constructing a risk model and nomogram for long non-coding RNAs that co-expressed with SUMF1

Data collected from the TCGA was subjected to Spearman analysis to identify long non-coding RNAs that co-expressed with SUMF1 in glioma tissues. Long non-coding RNAs exhibiting a correlation coefficient whose absolute value exceeded 0.6 were categorized as strongly correlated with SUMF1. To evaluate the relationship between SUMF1, the 13 co-expressed long non-coding RNAs (LINC01426, AC061992.2, CARD8-AS1, AC083855.2, AC083799.1, AC027307.2, AC026356.1, LYRM4-AS1, WAKMAR2, LINC01852, AC083837.1, ZNNT1, and LINC02636), and poor prognosis in glioma patients, we performed a Lasso regression analysis. The significance levels were represented in nomogram, and risk models were constructed accordingly.

### The relationship between SUMF1 expression and the immune microenvironment

We performed estimate and single-sample gene set enrichment analysis (ssGSEA) of glioma tissue data obtained from the TCGA to evaluate the immune microenvironment within glioma [18]. The integration of SUMF1 gene expression data with immune scoring data was accomplished with Perl language. The relationships between SUMF1 gene levels, immune cells, and immune, estimate, and stromal scores were subsequently investigated through Spearman correlation analysis. Additionally, we analyzed the expression levels of SUMF1 across various groups that were stratified by immune cells and immune, estimate, and stromal scores.

### The relationship between SUMF1 expression and immune cell markers

The names of the genes for immune cell markers were acquired based on previously published literature [17]. Using the Perl programming language, we obtained the expression data for the SUMF1 gene and cell markers in glioma tissues from the TCGA database. Subsequently, we conducted Spearman correlation analysis to investigate the relationship between the expression level of the SUMF1 gene and immune cell markers.

### Gene expression profiling interactive analysis (GEPIA) database

The GEPIA database incorporated cancer patient data from the TCGA and normal tissue data from healthy individuals in the GTEx database. Within the GEPIA database, we used the correlation module to investigate the correlation between SUMF1 levels and immune infiltrating cell marker levels in glioma.

### Constructing the cell models for inhibiting SUMF1 expression

Glioma U118 and U251 cells were cultured in DMEM medium (China), which contained 10% fetal bovine serum. Following the protocol provided by the supplier, siRNA transfection was performed under optimal conditions for glioma cell growth. The siRNA targeting SUMF1 gene was designed and synthesized by Genepharma (China). The forward sequence of SUMF1 was 5′–3′ GCGACTCCTTGTCTTTGAT, and the reverse sequence was TCAAAGACAAAGGAGTCGCT. Total RNAs and proteins from control cells and inhibiting SUMF1 expression cells were collected. We subsequently performed 24 h transfection. SUMF1 mRNA and protein expression levels were determined using standard RT-PCR and western blotting [17, 19].

### Western blotting

Glioma cell proteins were extracted using cell lysis buffer to disrupt the cell membranes and release the proteins. The protein sample was separated using gel electrophoresis and subsequently transferred from the gel to a polyvinylidene fluoride (PVDF) membrane. The proteins were then incubated with the 1:1000 snail, SUMF1, and vimentin (Proteintech, China). Following multiple washings, the secondary antibody was applied. The addition of color substrate facilitated color development and exposure.

### Cell proliferation

After counting the U118 and U251 cells, they were plated into a 96-well plate, with 3000 cells per well. Once the glioma cells were attached to the wall, 10 ul of CCK-8 solution was added, shaken, and then incubated in a thermostatic box for 2 h. The absorbances of each well were then measured with an enzyme label meter. Additional measurements were obtained 24, 48, 72, and 96 h later using the same method.

### Cell migration using wound healing

Glioma cells were digested in the logarithmic growth phase with trypsin to obtain single-cell suspension. They were then seeded in a 6-well plate for cultivation. After overnight incubation when the confluence reached approximately 90%, the scratch was made using a 200-μl pipette tip. The cells were washed thrice with phosphate buffer solution (PBS) to remove the scraped cells, and images were obtained. Another photograph was taken 24 h later. The ImageJ software was used for data processing to calculate the migration rate of glioma cells.

### Cell migration and invasion using a transwell assay

Glioma cells were digested in the growth phase with trypsin to obtain a single-cell suspension. After the cell density was adjusted to 1 × 10^5^/ml, 600 μl of the culture medium containing fetal bovine serum was added to the lower chamber of a 24-well plate. A transwell chamber was placed in the well, and 200 μl of serum-free medium containing cells was added to the upper chamber. The plate was then placed in an incubator and cultured for 24 h. For the invasion experiment, a matrix gel was prepared in advance. The ImageJ software was used for data processing to calculate the migration and invasion rates of glioma cells.

### Statistical analyses

The expression of SUMF1 in the TCGA and XENA databases was assessed with a Wilcoxon rank-sum test, while the results of CCK-8, wound healing, transwell, and other studies were evaluated with the *t* test. The chi-square test was used to analyze the relationship between SUMF1 levels and clinical characteristics of the glioma patients. The relationship between SUMF1 expression and various aspects of patient prognosis, diagnosis, and the immune microenvironment in glioma were explored using ROC, survival, ssGSEA, estimate, and Spearman methods. The statistical significance of cell experiments was set to *P* < 0.05.

### Data availability

The datasets used in this study are accessible through the TCGA, GTEx, XENA, and GEPIA databases, and research data can be obtained from corresponding authors.

## RESULTS

### SUMF1 expression was significantly increased in glioma

**Figure 1:**
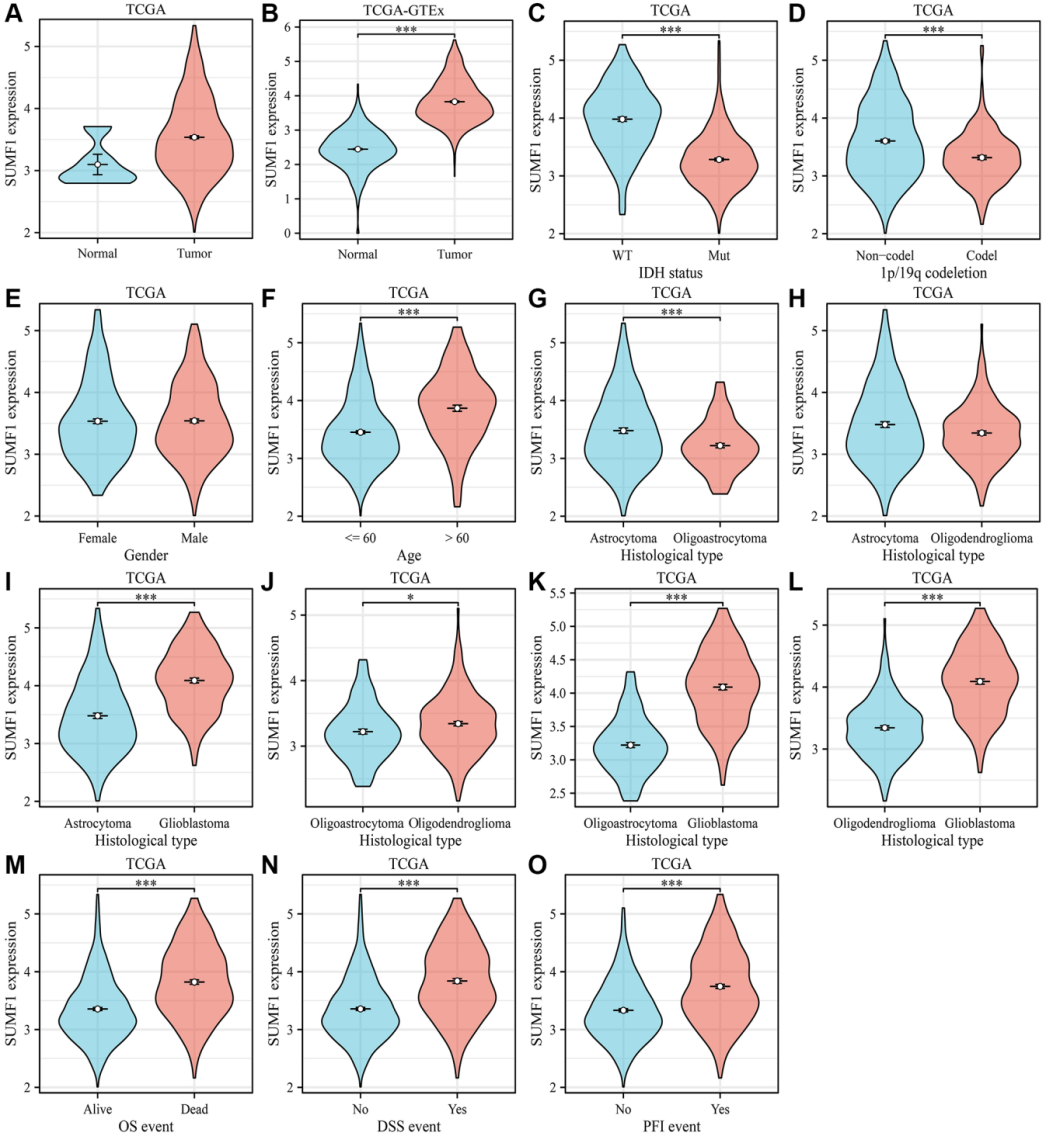
**SUMF1 expression levels in glioma.** (**A**) Normal vs. glioma in TCGA database; (**B**) Normal vs. glioma in the XNEA database; (**C**) Wild-type vs. mutant in IDH status; (**D**) Non-codel vs. codel in 1p/19q codeletion; (**E**) Female vs. Male in sex; (**F**) ≤60 vs. >60 in age; (**G**) Astrocytoma vs. oligoastrocytoma; (**H**) Astrocytoma vs. oligodendroglioma; (**I**) Astrocytoma vs. glioblastoma; (**J**) Oligoastrocytoma vs. oligodendroglioma; (**K**) Oligoastrocytoma vs. glioblastoma; (**L**) Oligodendroglioma vs. glioblastoma; (**M**) Alive vs. dead in OS; (**N**, **O**) No vs. yes in DSS and PFI. Abbreviations: DSS: disease-specific survival; IDH: isocitrate dehydrogenase; OS: overall survival; PFI: progression-free interval.

The levels of SUMF1 were significantly higher in glioma tissues in the TCGA and XNEA databases than in normal brain tissues (Figure 1A, 1B). Likewise, glioma tissues with the IDH wild-type, 1p/19q codeletion (non-codel), and those collected from individuals aged >60 years exhibited an overexpression of SUMF1 (Figure 1C–1F). SUMF1 was downregulated in oligoastrocytoma and oligodendroglioma tissues and overexpressed in glioblastoma tissues relative to astrocytoma tissues (Figure 1G–1I). Oligodendroglioma and glioblastoma tissues exhibited more SUMF1 than did oligoastrocytoma tissues (Figure 1J, 1K). Glioblastoma tissues exhibited a higher expression of SUMF1 than did oligodendroglioma tissues (Figure 1L). In relation to samples obtained from alive patients with DSS, OS, and PFI events, tissues from deceased patients exhibited overexpressed SUMF1 (Figure 1M–1O).

### SUMF1 overexpression was correlated with poor prognosis, IDH status, 1p/19q codeletion, age, and histological subtype in patients with glioma

Significant correlations were observed between SUMF1 overexpression and various factors including IDH status (wild-type or mutant), 1p/19q codeletion, age (≤60 or >60), histological type (astrocytoma, oligoastrocytoma, oligodendroglioma, or glioblastoma), OS event (alive or deceased), DSS event (alive or deceased), and PFI event (alive or deceased). No significant correlation was found between SUMF1 overexpression and 1p/19q codeletion and gender (Table 1).

**Table 1:** Correlation between the overexpression of SUMF1 and adverse features in patients with glioma.

**Characteristics**	**Down-expression of SUMF1**	**High expression of SUMF1**	***P*-value**
**IDH status**			
WT	44 (6.4%)	202 (29.3%)	<0.001
Mut	302 (43.8%)	141 (20.5%)	
**1p/19q codeletion**			
Non-codel	238 (34.4%)	282 (40.8%)	<0.001
Codel	111 (16%)	61 (8.8%)	
**Gender**			
Female	152 (21.7%)	146 (20.9%)	0.623
Male	197 (28.2%)	204 (29.2%)	
**Age**			
≤60	314 (44.9%)	242 (34.6%)	<0.001
>60	35 (5%)	108 (15.5%)	
**Histological type**			
Astrocytoma	106 (15.2%)	90 (12.9%)	<0.001
Oligoastrocytoma	100 (14.3%)	35 (5%)	
Oligodendroglioma	122 (17.5%)	78 (11.2%)	
Glioblastoma	21 (3%)	147 (21%)	
**Overall survival event**			
Alive	265 (37.9%)	162 (23.2%)	<0.001
Dead	84 (12%)	188 (26.9%)	
**DSS event**			
No	269 (39.7%)	165 (24.3%)	<0.001
Yes	73 (10.8%)	171 (25.2%)	
**PFI event**			
No	222 (31.8%)	131 (18.7%)	<0.001
Yes	127 (18.2%)	219 (31.3%)	

Abbreviations: DSS: disease-specific survival; IDH: isocitrate dehydrogenase; PFI: progression-free interval.

### SUMF1 overexpression was associated with a diagnosis of glioma and poor prognosis for afflicted patients

**Figure 2:**
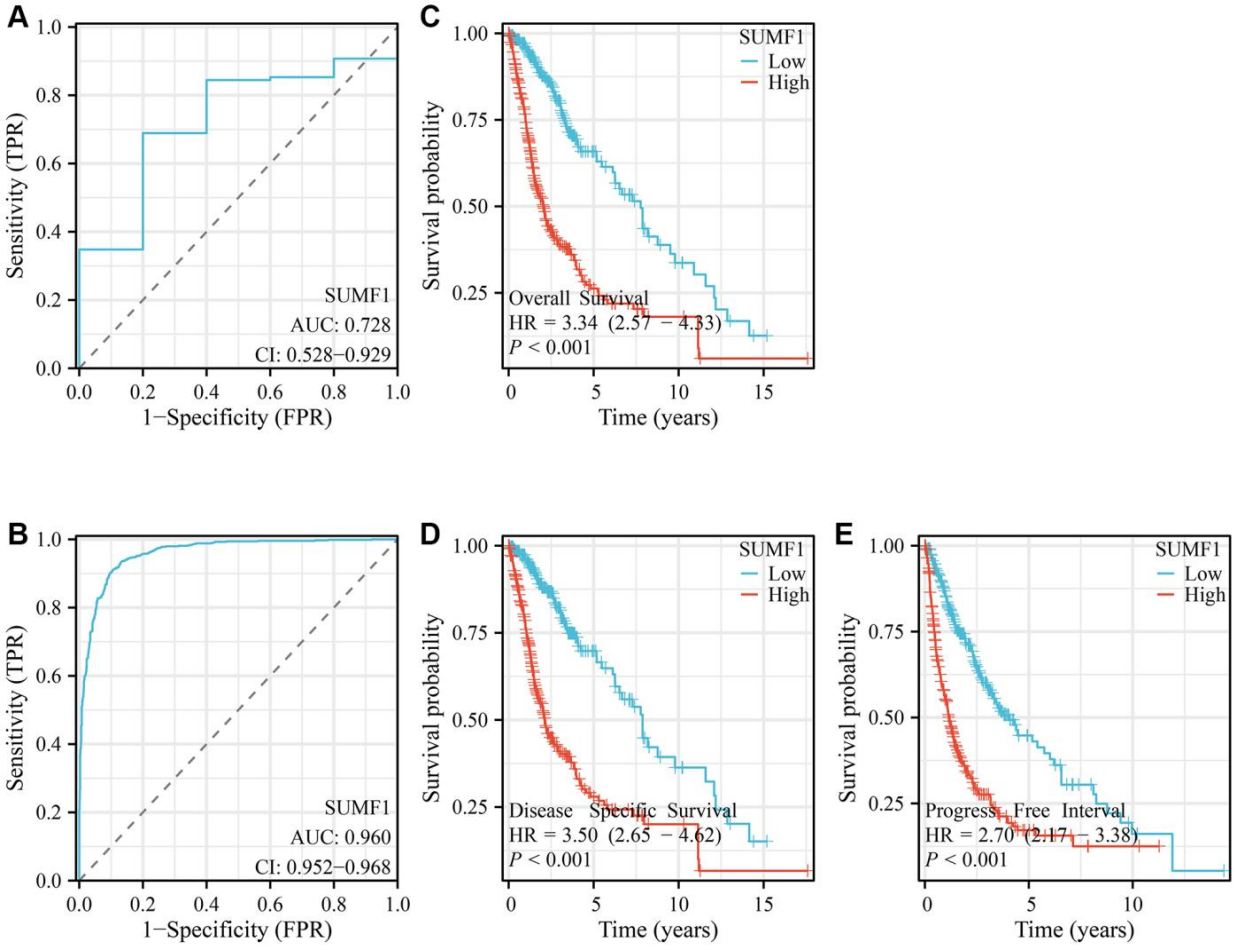
**SUMF1 overexpression can be used as a diagnostic and prognostic tool in the treatment of glioma.** (**A**) The AUC of SUMF1 from the TCGA database; (**B**) The AUC of SUMF1 from the XNEA database; (**C**–**E**) SUMF1 overexpression is predictive of poor prognosis.

The analysis of the TCGA data revealed an AUC value of 0.728 for SUMF1 expression in glioma tissues (Figure 2A). Similarly, the analysis of the XENA data yielded an AUC of 0.96 (Figure 2B). The survival analysis indicated a poorer prognosis for glioma patients with an overexpression of SUMF1 (Figure 2C–2E). Furthermore, the time-dependent ROC analysis showed that the overexpression of SUMF1 had significant predictive value for patients with glioma at the 1-, 3-, and 5-year time points (Supplementary Figure 1).

### Overexpression of SUMF1 was strongly associated with unfavorable prognosis in subgroups of patients with glioma

**Figure 3:**
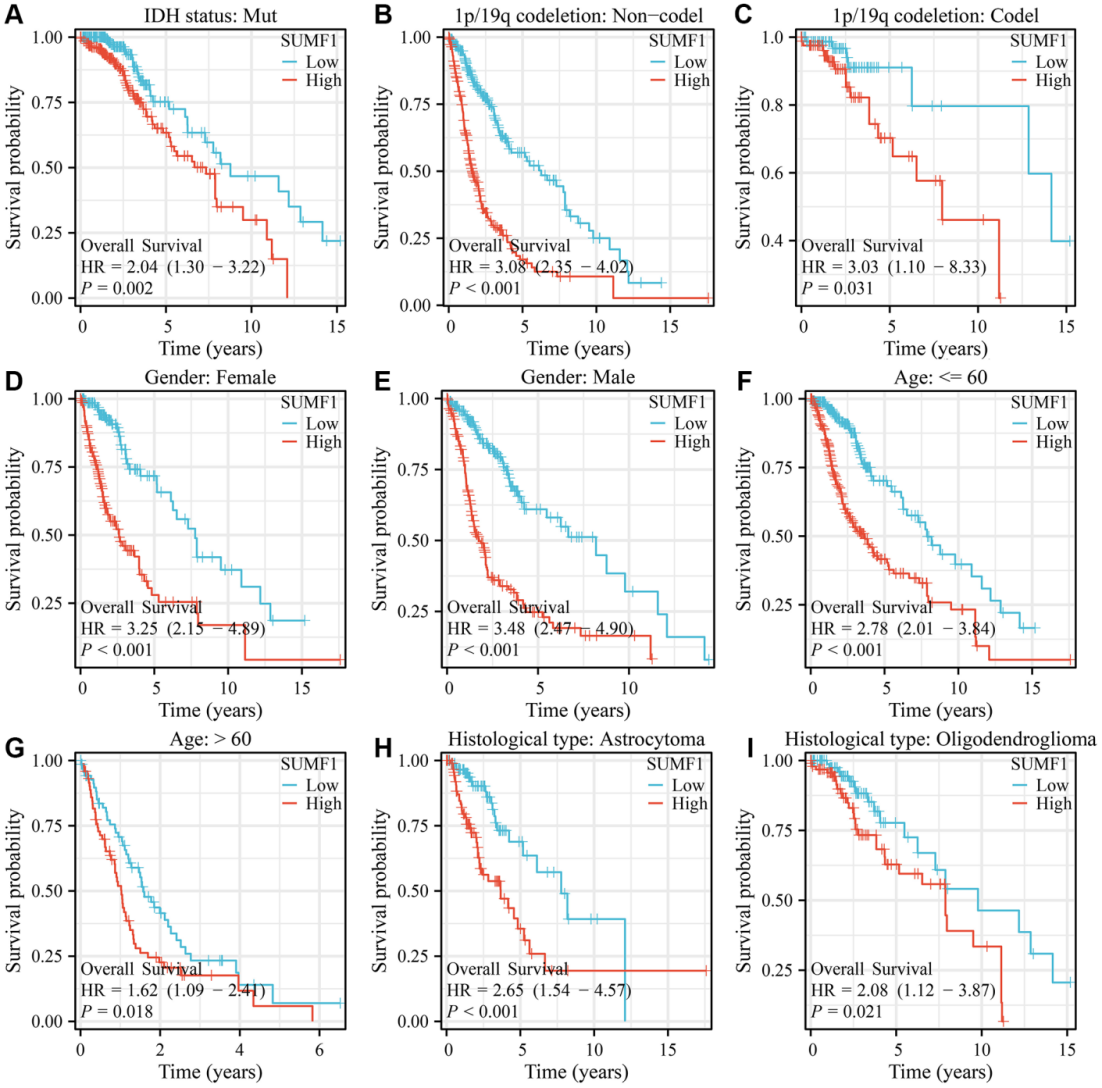
**SUMF1 overexpression is significantly correlated with poor survival time in subgroups of glioma.** (**A**) IDH mutant patients; (**B**) Patients with the non-codel in 1p/19q codeletion; (**C**) Patients with the codel in 1p/19q codeletion; (**D**) Female; (**E**) Male; (**F**) Age ≤60; (**G**) Age >60; (**H**) Astrocytoma; (**I**) Oligodendroglioma.

**Figure 4:**
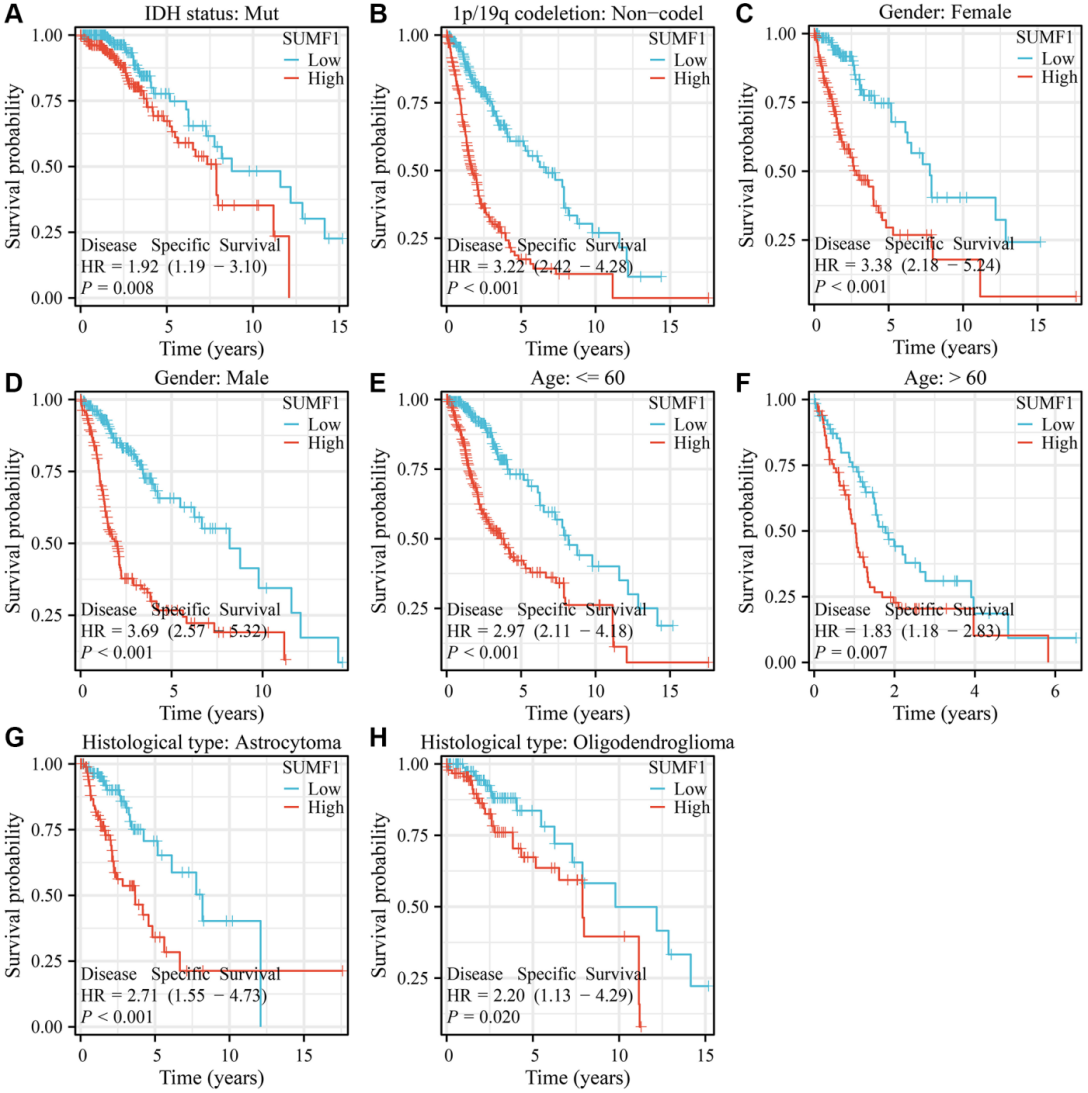
**SUMF1 overexpression is significantly correlated with poor DSS in subgroups of with glioma.** (**A**) IDH mutant patients; (**B**) Patients with the non-codel in 1p/19q codeletion; (**C**) Female; (**D**) Male; (**E**) Age ≤60; (**F**) Age >60; (**G**) Astrocytoma; (**H**) Oligodendroglioma.

**Figure 5:**
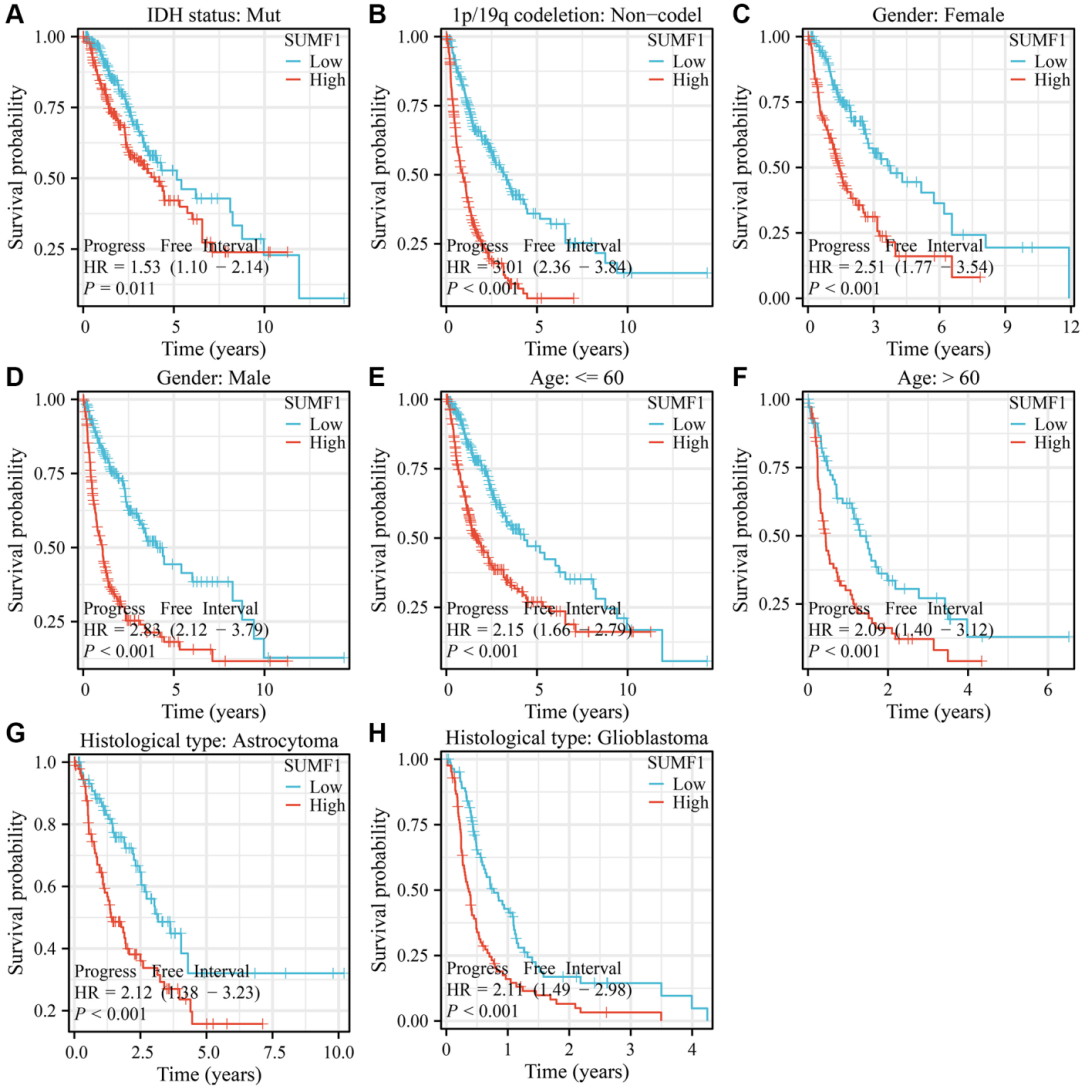
**SUMF1 overexpression is significantly correlated with cancer progression in subgroups of patients with glioma.** (**A**) IDH mutant patients; (**B**) The patients with the non-codel in 1p/19q codeletion; (**C**) Female; (**D**) Male; (**E**) Age ≤60; (**F**) Age >60; (**G**) Astrocytoma; (**H**) Oligodendroglioma.

For glioma with the IDH mutation, 1p/19q codeletion (codel or non-codel), gender (female or male), age (>60 or ≤60), and type of glioma (astrocytoma or oligodendroglioma), a significant correlation was observed between the overexpression of SUMF1 and adverse survival outcomes in patients (Figure 3). Furthermore, in glioma with the IDH mutation, 1p/19q non-codel, gender (female or male), age (>60 or ≤60), and type of glioma (astrocytoma or oligodendroglioma), there was also a noteworthy association between the overexpression of SUMF1 and adverse outcomes relating to DSS and disease progression in patients (Figures 4, 5).

### Overexpression of SUMF1 served as an independent risk factor for a poor prognosis among patients with glioma

**Figure 6:**
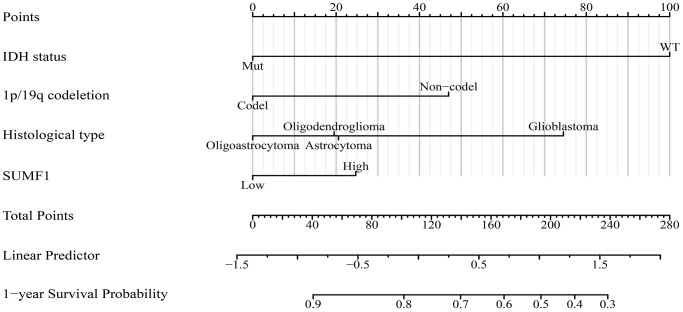
**SUMF1-related nomogram of PFI for glioma patients.** Abbreviations: PFI: progression-free interval.

The Cox regression analysis revealed that several factors, including the overexpression of SUMF1, IDH status, 1p/19q codeletion, age, and histological subtype, contribute to the risk of poor OS, DSS, and cancer progression among glioma patients (Tables 2–4). Specifically, the overexpression of SUMF1 emerged as an independent risk factor for cancer progression (Table 4). To further explore the relationship between SUMF1 overexpression and patient outcomes, the nomogram was constructed to reflect the association of SUMF1 overexpression, IDH status, 1p/19q codeletion, age, and histological subtype with PFI based on a *P* < 0.05 (Figure 6).

**Table 2:** Risk factors for poor survival among patients with glioma.

**Characteristics**	**Total (*N*)**	**HR (95% CI)**	***P*-value**	**HR (95% CI)**	***P*-value**
**IDH status**	688		<0.001		
WT	246	Reference		Reference	
Mut	442	0.116 (0.089–0.151)	<0.001	0.271 (0.180–0.406)	<0.001
**1p/19q codeletion**	691		<0.001		
Non-codel	520	Reference		Reference	
Codel	171	0.225 (0.147–0.346)	<0.001	0.690 (0.397–1.197)	0.187
**Gender**	698		0.071		
Female	297	Reference			
Male	401	1.250 (0.979–1.595)	0.073		
**Age**	698		<0.001		
≤60	555	Reference		Reference	
>60	143	4.696 (3.620–6.093)	<0.001	1.568 (1.156–2.128)	0.004
**Histological type**	698		<0.001		
Astrocytoma	196	Reference		Reference	
Oligoastrocytoma	135	0.646 (0.412–1.013)	0.057	0.917 (0.575–1.461)	0.714
Oligodendroglioma	199	0.578 (0.393–0.849)	0.005	0.786 (0.505–1.225)	0.288
Glioblastoma	168	6.791 (4.931–9.352)	<0.001	2.534 (1.739–3.692)	<0.001
**SUMF1**	698		<0.001		
Low	348	Reference		Reference	
High	350	3.338 (2.573–4.331)	<0.001	1.222 (0.891–1.678)	0.214

**Table 3:** Risk factors for disease-specific survival among patients with glioma.

**Characteristics**	**Total (*N*)**	**HR (95% CI)**	***P*-value**	**HR (95% CI)**	***P*-value**
**IDH status**	667		<0.001		
WT	232	Reference		Reference	
Mut	435	0.110 (0.083–0.145)	<0.001	0.239 (0.156–0.366)	<0.001
**1p/19q codeletion**	671		<0.001		
Non-codel	501	Reference		Reference	
Codel	170	0.200 (0.125–0.320)	<0.001	0.653 (0.359–1.186)	0.161
**Gender**	677		0.104		
Female	289	Reference			
Male	388	1.236 (0.956–1.599)	0.107		
**Age**	677		<0.001		
≤60	544	Reference		Reference	
>60	133	4.528 (3.430–5.978)	<0.001	1.471 (1.068–2.027)	0.018
**Histological type**	677		<0.001		
Astrocytoma	193	Reference		Reference	
Oligoastrocytoma	133	0.593 (0.368–0.957)	0.033	0.866 (0.527–1.423)	0.570
Oligodendroglioma	196	0.540 (0.361–0.809)	0.003	0.760 (0.479–1.207)	0.245
Glioblastoma	155	6.602 (4.739–9.197)	<0.001	2.494 (1.692–3.674)	<0.001
**SUMF1**	677		<0.001		
Low	341	Reference		Reference	
High	336	3.497 (2.650–4.615)	<0.001	1.126 (0.795–1.594)	0.503

**Table 4:** Risk factors for PFI among patients with glioma.

**Characteristics**	**Total (*N*)**	**HR (95% CI)**	***P*-value**	**HR (95% CI)**	***P*-value**
**IDH status**	688		<0.001		
WT	246	Reference		Reference	
Mut	442	0.150 (0.118–0.190)	<0.001	0.296 (0.211–0.416)	<0.001
**1p/19q codeletion**	691		<0.001		
Non-codel	520	Reference		Reference	
Codel	171	0.296 (0.214–0.410)	<0.001	0.558 (0.363–0.857)	0.008
**Gender**	698		0.525		
Female	297	Reference			
Male	401	1.072 (0.865–1.328)	0.525		
**Age**	698		<0.001		
≤60	555	Reference		Reference	
>60	143	2.892 (2.283–3.664)	<0.001	1.056 (0.795–1.403)	0.707
**Histological type**	698		<0.001		
Astrocytoma	196	Reference		Reference	
Oligoastrocytoma	135	0.567 (0.394–0.816)	0.002	0.774 (0.533–1.126)	0.180
Oligodendroglioma	199	0.634 (0.466–0.862)	0.004	0.988 (0.687–1.422)	0.950
Glioblastoma	168	4.412 (3.350–5.810)	<0.001	1.905 (1.368–2.655)	<0.001
**SUMF1**	698		<0.001		
Low	348	Reference		Reference	
High	350	2.705 (2.166–3.377)	<0.001	1.357 (1.045–1.762)	0.022

### Functions of SUMF1 co-expressed genes

The 584 co-expressed genes with SUMF1 were significantly enriched in various biological processes including glycoprotein metabolism, T-cell activation, leukocyte-cell adhesion, cell differentiation, lymphocyte proliferation, leukocyte proliferation, T-cell differentiation, T-cell-mediated cytotoxicity, apoptosis, and others (Supplementary Table 1).

### A correlation exists between the risk score calculated from the long non-coding RNAs that are co-expressed with SUMF1 and the prognosis of patients with glioma

We found 13 long non-coding RNAs LINC01426, AC061992.2, CARD8-AS1, AC083855.2, AC083799.1, AC027307.2, AC026356.1, LYRM4-AS1, WAKMAR2, LINC01852, AC083837.1, ZNNT1, and LINC02636 of co-expressed with SUMF1. The Lasso analysis revealed that the expression of LINC01426, AC061992.2, CARD8-AS1, AC083855.2, AC027307.2, AC026356.1, LYRM4-AS1, WAKMAR2, and LINC02636 were associated with adverse survival time and DSS in patients with glioma. Moreover, a risk model constructed based on this analysis was indicative of poor prognosis among patients with glioma (Supplementary Figure 2A, 2B). Additionally, the expression of LINC01426, AC061992.2, CARD8-AS1, AC083855.2, AC027307.2, WAKMAR2, LINC02636, and SUMF1 were correlated with adverse PFI in patients with glioma. The risk model established from this analysis confirmed the association with prognosis among patients with glioma (Supplementary Figure 2C). A nomogram shows the relationship between the expression of the long non-coding RNAs and SUMF1 with respect to the PFI in patients with glioma (Supplementary Figure 3).

### Inhibiting SUMF1 expression could deter the growth and metastasis of glioma cells

**Figure 7:**
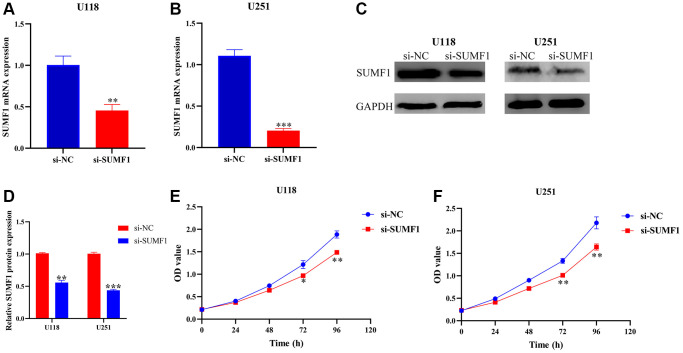
**Inhibiting the expression of SUMF1 deters the proliferation of glioma cells.** (**A**–**D**) Cell models using RT-PCR and Western blotting; (**E**, **F**) Cell proliferation using CCK-8.

**Figure 8:**
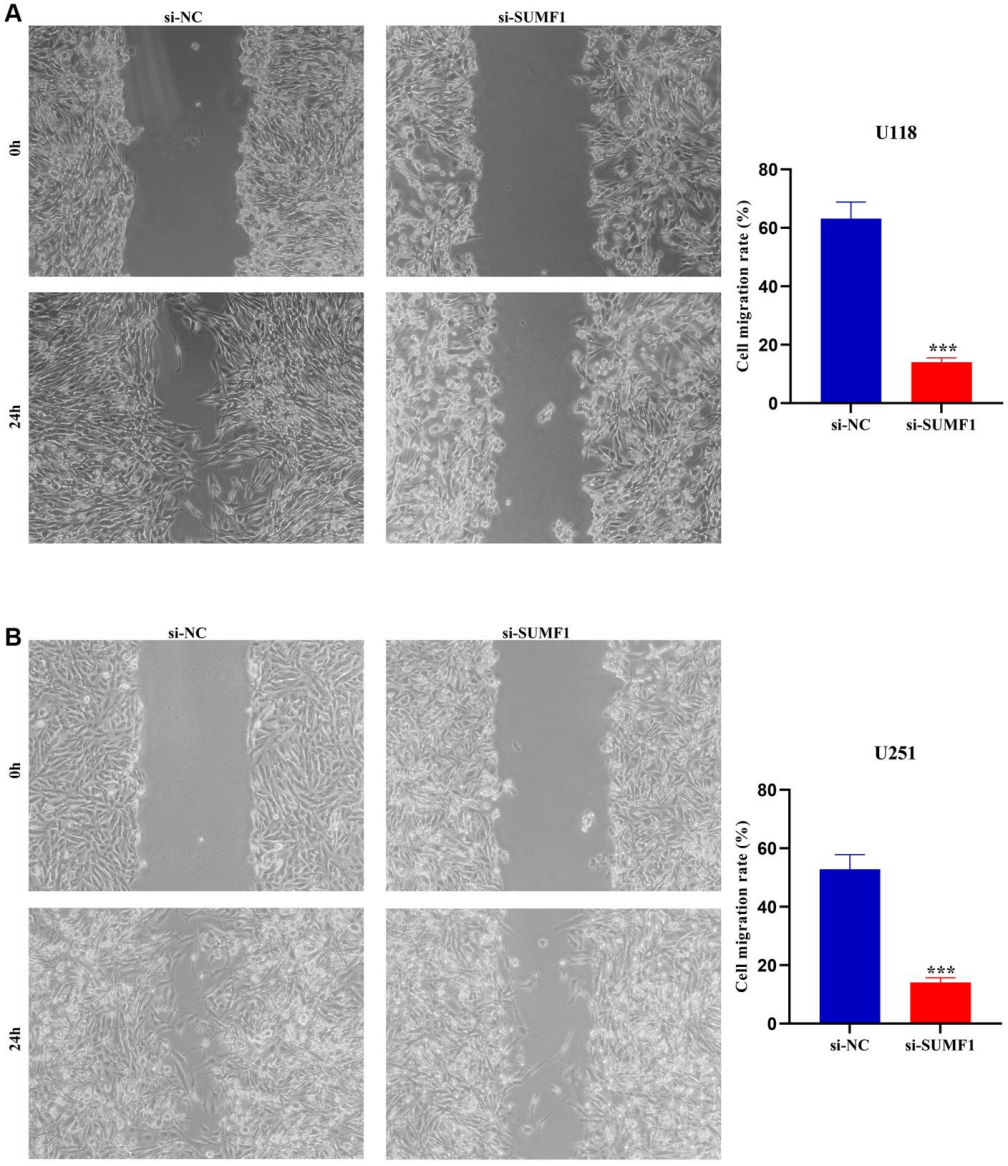
**Wound healing shows that inhibiting the expression of SUMF1 deters the migration of glioma cells.** (**A**) U118 cells; (**B**) U251 cells.

**Figure 9:**
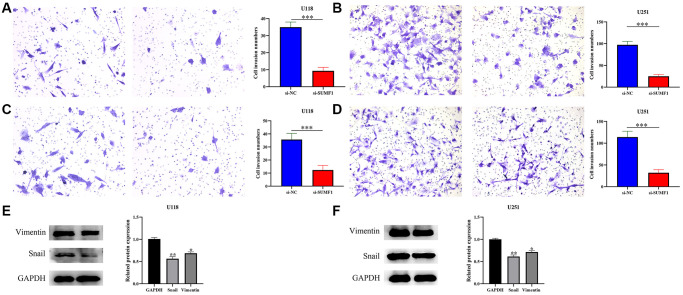
**Inhibiting the expression of SUMF1 deters the migration and invasion of glioma cells through EMT.** (**A**, **B**) Cell migration; (**C**, **D**) Cell invasion; (**E**, **F**) Snail and vimentin protein expression in glioma cells that inhibit SUMF1 expression.

The results of the RT-PCR and Western blotting showed that successfully constructed a cell models (Figure 7A–7C). The CCK-8 showed a significant statistical difference in the absorbance values of U251 and U118 between 72 and 96 h (Figure 7D–7F), suggesting that inhibiting SUMF1 expression inhibits U251 and U118 cell growth in glioma. The results of the wound healing and transwell tests showed that inhibiting SUMF1 expression could inhibit the U251 and U118 cell migration and invasion in glioma (Figures 8 and 9A–9D). The results of Western blotting showed that inhibiting SUMF1 expression can inhibit epithelial mesenchymal transition (EMT) and thus decrease snail and vimentin protein expression (Figure 9E, 9F).

### Overexpression of SUMF1 is correlated with the immune cells and scores in glioma

**Figure 10:**
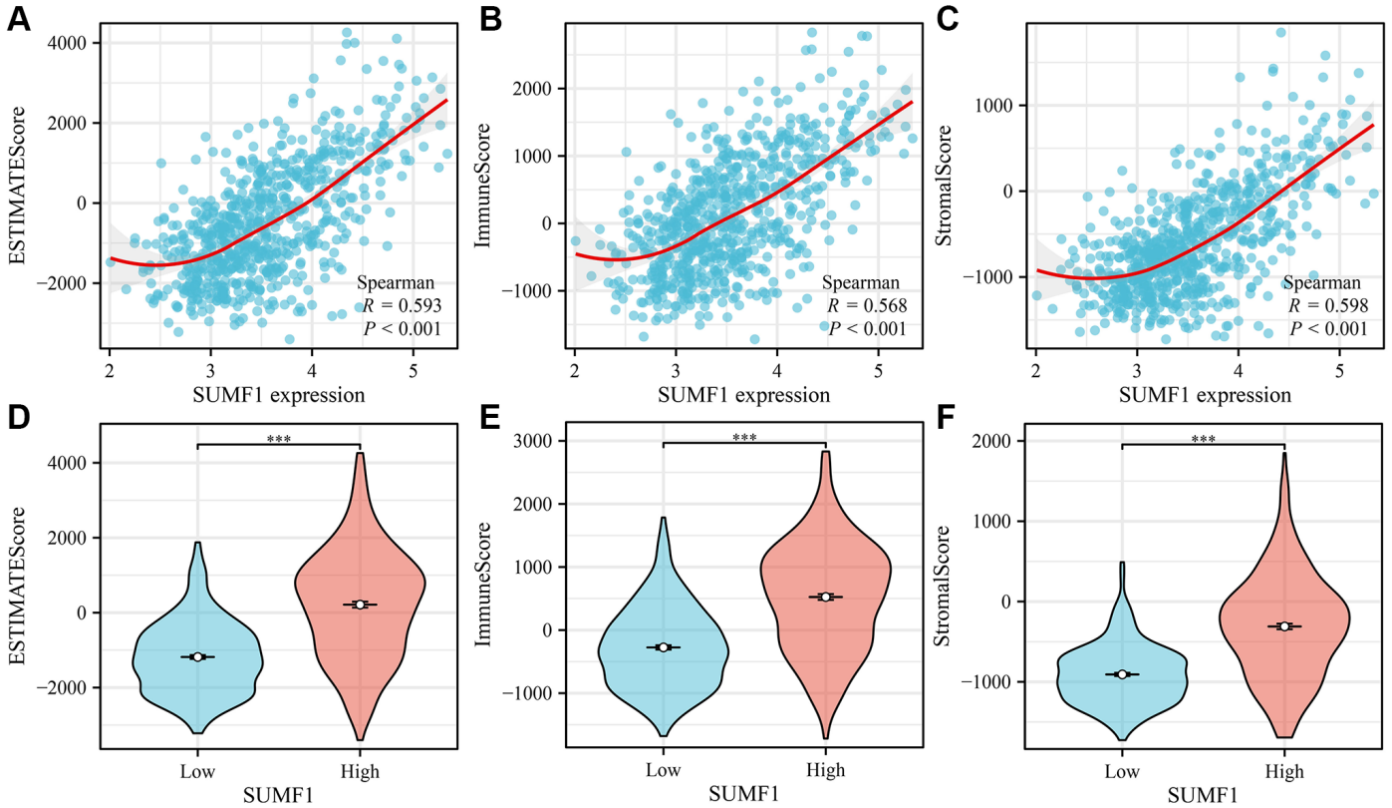
**Correlation between SUMF1 and glioma immunity.** (**A**–**C**) Correlation analysis; (**D**–**F**) Expression analysis after grouping.

**Figure 11:**
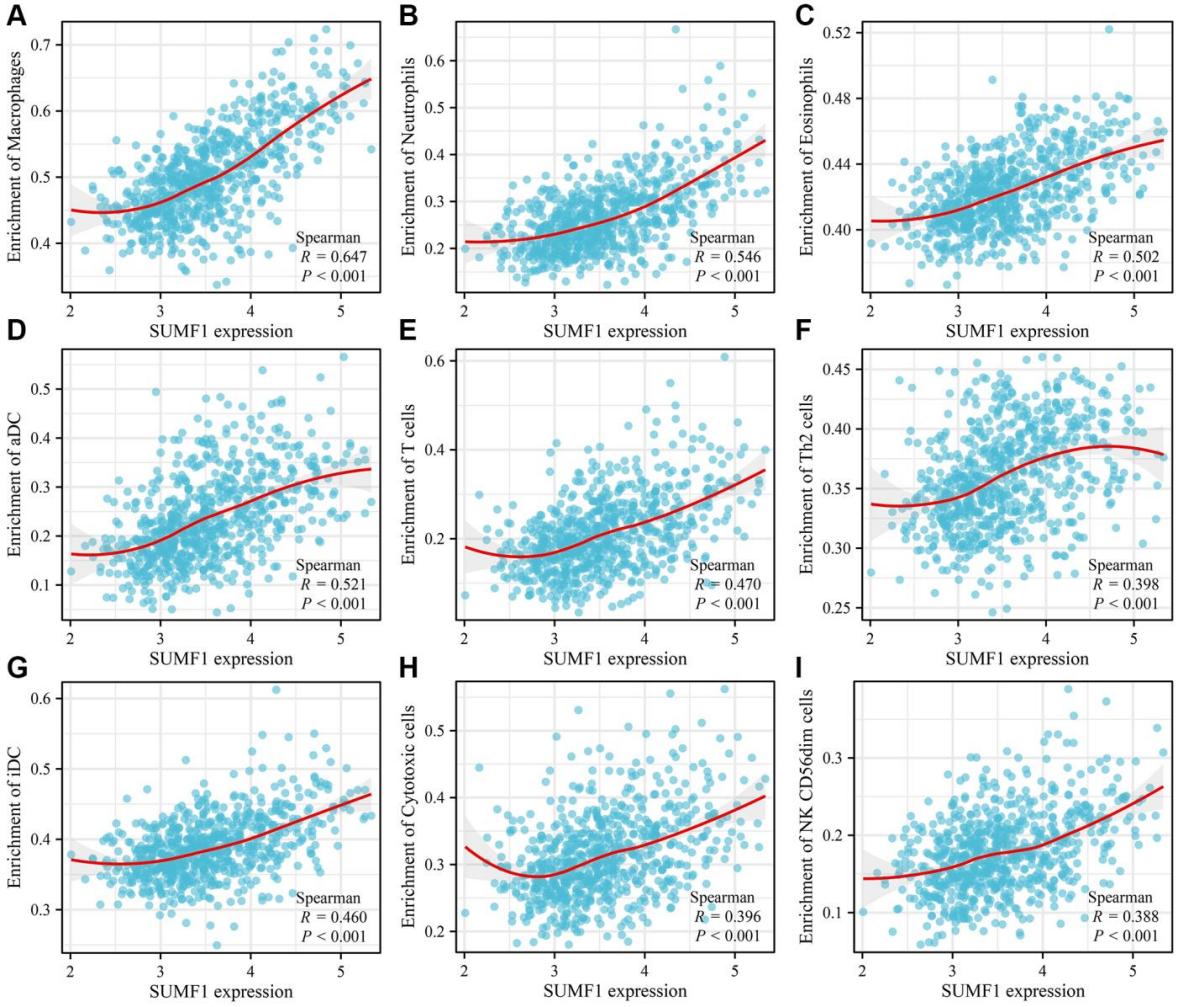
**Correlation between SUMF1 and glioma immune cells.** (**A**) Macrophages; (**B**) Neutrophils; (**C**) Eosinophils; (**D**) aDC; (**E**) T cells; (**F**) Th2 cells; (**G**) iDC; (**H**) Cytotoxic cells; (**I**) NK CD56dim cells.

Based on the analysis of TCGA data, the expression of SUMF1 was significantly correlated with various immune cell types and scores. Specifically, there was a positive correlation between SUMF1 overexpression and immune (r = 0.568), stromal (r = 0.598), and ESTIMATE (r = 0.593) scores (Figure 10A–10C). The immune, stromal, and ESTIMATE scores significantly differed between the high- and low-SUMF1 expression groups (Figure 10D–10F). There was a positive correlation with aDC (r = 0.521), cytotoxic cells (r = 0.396), DC (r = 0.188), eosinophils (r = 0.502), Th1 cells (r = 0.096), iDC (r = 0.460), macrophages (r = 0.647), neutrophils (r = 0.546), NK cells (r = 0.159), T cells (r = 0.470), T helper cells (r = 0.197), Th17 cells (r = 0.348), and Th2 cells (r = 0.398). However, we found a negative correlation with CD8 T cells (r = −0.118), NK CD56bright cells (r = −0.163), pDC (r = −0.375), Tcm (r = −0.244), Tem (r = −0.113), TFH (r = −0.191), and Tgd (r = −0.330; Figure 11). The immune cell types differ significantly between high- and low-SUMF1 expression groups (Supplementary Figure 4). Overall, these results suggested a strong association between SUMF1 expression and immune microenvironment, emphasizing the potential role of SUMF1 in the immune response.

### SUMF1 overexpression is correlated with the presence of immune cell markers in glioma

SUMF1 expression was significantly correlated with the levels of the immune genes CD1C (r = 0.465), STAT6 (r = 0.4), STAT1 (r = 0.525), STAT3 (r = 0.601), LAG3 (r = 0.238), IL10 (r = 0.533), CSF1R (r = 0.288), CD163 (r = 0.575), CD68 (r = 0.159), ITGAM (r = 0.429), HLA-DRA (r = 0.667), PTGS2 (r = 0.245), HLA-DPB1 (r = 0.646), STAT5A (r = 0.538), NRP1 (r = 0.594), IFNG (r = 0.333), CCR7 (r = 0.523), HLA-DQB1 (r = 0.594), GZMB (r = 0.457), IL13 (r = −0.121), CD19 (r = 0.39), HLA-DPA1 (r = 0.639), CD3E (r = 0.578), MS4A4A (r = 0.578), CD3D (r = 0.607), GATA3 (r = 0.507), CD86 (r = 0.526), CD8B (r = 0.542), IL21 (r = 0.164), KIR3DL2 (r = 0.298), KIR2DS4 (r = 0.241), NOS2 (r = 0.080), ITGAX (r = 0.322), KIR3DL1 (r = 0.159), KIR2DL4 (r = 0.317), CCL2 (r = 0.498), HAVCR2 (r = 0.543), TGFB1 (r = 0.528), KIR2DL1 (r = 0.183), PDCD1 (r = 0.513), CD8A (r = 0.373), IRF5 (r = 0.468), CTLA4 (r = 0.404), KIR2DL3 (r = 0.241), FOXP3 (r = 0.203), VSIG4 (r = 0.480), CCR8 (r = 0.328), TBX21 (r = 0.386), CD2 (r = 0.616), and CD79A (r = 0.215) in the Table 5. The association between the expression of the SUMF1 gene and the immune genes listed above has been confirmed by the TIMER database (Table 5).

**Table 5:** A correlation between SUMF1 and glioma immune cell markers in the TCGA and GEPIA databases.

**Markers**	**TCGA**			**GEPIA**	
	**Coefficient**	***P*-value**		**Coefficient**	***P*-value**
**HLA-DRA**	0.666963992	2.13088E-91		0.62	9.5e-73
**HLA-DPB1**	0.645677045	6.25405E-84		0.59	5.8e-66
**HLA-DPA1**	0.638680534	1.32481E-81		0.6	7.9e-68
**CD2**	0.615569309	2.47152E-74		0.56	2.6e-58
**CD3D**	0.606824765	9.75628E-72		0.53	5.4e-50
**STAT3**	0.601087926	4.45862E-70		0.55	5.9e-54
**HLA-DQB1**	0.594344498	3.61285E-68		0.43	4.7e-32
**NRP1**	0.593709478	5.43582E-68		0.53	3.3e-50
**CD3E**	0.578242051	8.65972E-64		0.53	1.4e-49
**MS4A4A**	0.577669122	1.22707E-63		0.51	7.1e-47
**CD163**	0.575365906	4.94728E-63		0.51	1.4e-45
**HAVCR2**	0.542926233	5.43476E-55		0.5	4.2e-44
**CD8B**	0.54169819	1.05332E-54		0.3	7.9e-16
**STAT5A**	0.538437956	6.0225E-54		0.5	7.3e-44
**IL10**	0.533302718	9.03249E-53		0.49	4e-43
**TGFB1**	0.528230119	1.25261E-51		0.44	2.5e-34
**CD86**	0.525709706	4.55078E-51		0.49	2.9e-42
**STAT1**	0.524991248	6.56044E-51		0.49	3e-42
**CCR7**	0.523217735	1.61221E-50		0.48	8.8e-41
**PDCD1**	0.513452633	2.07334E-48		0.44	5.1e-33
**GATA3**	0.507304089	4.07437E-47		0.44	4.3e-33
**CCL2**	0.497885138	3.47514E-45		0.46	5e-37
**VSIG4**	0.479557909	1.34876E-41		0.42	4.5e-31
**IRF5**	0.468402159	1.62336E-39		0.41	1.7e-29
**CD1C**	0.464715137	7.60879E-39		0.41	7.8e-29
**GZMB**	0.457198455	1.67483E-37		0.4	7.6e-28
**ITGAM**	0.428990754	9.41787E-33		0.39	8.9e-27
**CTLA4**	0.404280672	6.0529E-29		0.36	1.3e-22
**STAT6**	0.400212043	2.39339E-28		0.37	5.1e-24
**CD19**	0.389643326	7.79302E-27		0.3	4.2e-16
**TBX21**	0.386182258	2.3729E-26		0.33	6.3e-19
**CD8A**	0.373302261	1.33297E-24		0.38	7.1e-25
**IFNG**	0.332884762	1.33546E-19		0.26	2.8e-12
**CCR8**	0.327983106	4.82916E-19		0.32	1.8e-17
**ITGAX**	0.322275234	2.09541E-18		0.27	3.4e-13
**KIR2DL4**	0.31718582	7.55505E-18		0.28	1.1e-13
**KIR3DL2**	0.298341049	7.06563E-16		0.21	4.3e-08
**CSF1R**	0.287685316	7.96796E-15		0.25	3.5e-11
**PTGS2**	0.245191288	4.67572E-11		0.27	1.4e-12
**KIR2DL3**	0.240882171	1.03537E-10		0.2	7.3e-08
**KIR2DS4**	0.240561643	1.09777E-10		0.17	4.9e-06
**LAG3**	0.237687455	1.84839E-10		0.14	2e-04
**CD79A**	0.215223591	8.62516E-09		0.14	2e-04
**FOXP3**	0.202951115	5.9488E-08		0.21	6.6e-08
**KIR2DL1**	0.183495943	1.00207E-06		0.17	1.2e-05
**IL21**	0.164443914	1.20912E-05		0.15	7.8e-05
**KIR3DL1**	0.159026778	2.33806E-05		0.21	4.1e-08
**CD68**	0.158832172	2.39316E-05	C	0.53	2.3e-51
**NOS2**	0.080182876	0.033788721	C	0.053	0.17
**IL13**	−0.121291832	0.001293009	C	−0.14	4e-04

## DISCUSSION

Changes in gene expression play a significant role in the pathogenesis of cancer [7–12, 14, 19–21]. For instance, the overexpression of transmembrane protein 147 (TMEM147) was observed in patients with hepatocellular carcinoma, and elevated TMEM147 expression was linked to poor prognosis [14]. Similarly, alterations in gene expression were implicated in the development of glioma [20, 21]. With multiple genes influencing the prognosis of patients with glioma and leading to unfavorable outcomes, it is crucial to identify novel genes that could improve prognostic accuracy [20, 21]. In this study, we found that the gene encoding SUMF1 was overexpressed in glioma tissues, particularly in those obtained from patients with IDH wild-type and non-codel subtype, aged >60 years, specific histological subtypes, and had poor OS, DSS, and PFI outcomes. Furthermore, SUMF1 overexpression was significantly associated with adverse prognosis, IDH status, age, and histological subtypes in patients with glioma. Analysis of the TCGA and XENA data revealed AUC values of 0.728 and 0.96, respectively, for SUMF1 overexpression in glioma. Moreover, SUMF1 overexpression was a notable risk factor independently associated with poor prognosis in patients with glioma. We further found that the expression of SUMF1, LINC01426, AC061992.2, CARD8-AS1, AC083855.2, AC027307.2, WAKMAR2, and LINC02636, were associated with adverse progression in patients with glioma. A risk model could predict poor prognosis and assess the relationship between the progression and prognosis of glioma based on the co-expression of long non-coding RNAs and SUMF1. These findings suggest that SUMF1 overexpression can serve as a biomarker for diagnosis and poor prognosis in patients with glioma.

The mechanism such as EMT has been significantly implicated in the occurrence and development of glioma [22–25]. For example, the expression of ADAM metallopeptidase with thrombospondin type 1 motif 8 (ADAMTS8) was found to be significantly diminished in glioma. The high expression of ADAMTS8 is associated with high survival, and its overexpression in glioma cells could inhibit cancer cell survival, invasion, migration, and tumor growth *in vivo*. Further, ADAMTS8 could inhibit the expression of matrix metallopeptidase 2 (MMP2) and matrix metallopeptidase 9 (MMP9) proteins during EMT and promote cell apoptosis [22]. The expression of coagulation factor 2 thrombin receptor (F2R) was observed to be upregulated in glioma tissues, and the overexpression of F2R is associated with poor prognosis in patients with glioma. The overexpression of F2R could promote the proliferation and metastasis of glioma cells through EMT, thus promoting tumor growth *in vivo* [25]. We found that inhibiting SUMF1 expression could deter the growth, migration, and invasion of glioma cells. In addition, we found that inhibiting SUMF1 expression can diminish snail and vimentin protein expression. Preliminary evidence suggests that SUMF1 can affect the glioma progression through EMT.

Immunotherapy has received increasing attention in the literature and has shown significant promise in addressing glioma progression [26–30]. For example, Cloughesy et al., found that neoadjuvant programmed cell death 1 (PDCD1) immunotherapy combined with postoperative adjuvant therapy significantly prolonged OS in glioma patients. PDCD1 inhibitors were associated with the upregulation of T cells and interferon-gamma-related gene expression and enhanced both local and systemic anti-tumor immune responses [30]. This previous finding informed our decision to analyze the relationship between SUMF1 and the glioma immune microenvironment. We found that SUMF1 expression was significantly correlated with glioma immune score, stromal score, ESTIMATE score, immune cells (aDC, B cells, CD8 T cells, cytotoxic cells, T cells, Tgd, Th17 cells, Th2 cells, and others), and immune cell markers (CD19, CD86, CD8B, CCL2, PDCD1, CD8A, CTLA4, CD79A, and others). These findings implicate SUMF1 in glioma progression.

The present comprehensive analysis and cellular experiments confirm that SUMF1 is overexpressed in glioma and may thus play a significant role in their progression. The heightened expression of SUMF1 was also significantly correlated with cancer diagnosis, IDH status, age, histological subtype, and prognosis. The overexpression of SUMF1 was positively correlated with poor prognosis and immune microenvironment and was an independent risk factor for poor prognosis in patients with glioma. Inhibiting SUMF1 expression could deter the growth, migration, and invasion of glioma cells. These findings suggest the promise of SUMF1 as a biomarker for poor prognosis in glioma patients. Despite the above findings, the present study was subject to limitations. Collecting more glioma tissues would have helped improve our data concerning SUMF1 expression. The relationship between SUMF1 expression and the prognosis of glioma patients was explored. In addition, we will explore the functions and mechanisms of SUMF1 in immune cells and verify the interaction between SUMF1 and immune inflammatory factors in the future.

## Supplementary Materials

Supplementary Figures

Supplementary Table 1
