# Supplementary material for: SUMF1 overexpression promotes tumorous cell growth and migration and is correlated with the immune status of patients with glioma
Source: Aging (Albany NY). 2024 Mar 7;16(5):4699–722. doi: 10.18632/aging.205626 (PMC10968700; doi:10.18632/aging.205626)
Supplement: Supplementary Table 1 [file aging-16-205626-s002.docx]

**Supplementary Table 1. Functions of SUMF1 co-expressed genes.**

| Type | ID | Description | P value |
| --- | --- | --- | --- |
| BP | GO:0048002 | antigen processing and presentation of peptide antigen | 3.21615E-17 |
| BP | GO:0019882 | antigen processing and presentation | 3.96351E-16 |
| BP | GO:0019886 | antigen processing and presentation of exogenous peptide antigen via MHC class II | 2.44459E-14 |
| BP | GO:0002478 | antigen processing and presentation of exogenous peptide antigen | 6.09685E-14 |
| BP | GO:0019884 | antigen processing and presentation of exogenous antigen | 1.34863E-13 |
| BP | GO:0002495 | antigen processing and presentation of peptide antigen via MHC class II | 2.10249E-13 |
| BP | GO:0002504 | antigen processing and presentation of peptide or polysaccharide antigen via MHC class II | 5.43542E-13 |
| BP | GO:0002831 | regulation of response to biotic stimulus | 6.92173E-13 |
| BP | GO:0002396 | MHC protein complex assembly | 1.48769E-12 |
| BP | GO:0002501 | peptide antigen assembly with MHC protein complex | 1.48769E-12 |
| BP | GO:0043123 | positive regulation of I-kappaB kinase/NF-kappaB signaling | 7.05385E-12 |
| BP | GO:0002443 | leukocyte mediated immunity | 1.23758E-11 |
| BP | GO:0042060 | wound healing | 8.72962E-11 |
| BP | GO:0002399 | MHC class II protein complex assembly | 1.31409E-10 |
| BP | GO:0002503 | peptide antigen assembly with MHC class II protein complex | 1.31409E-10 |
| BP | GO:0051604 | protein maturation | 1.88026E-10 |
| BP | GO:0009100 | glycoprotein metabolic process | 7.80714E-10 |
| BP | GO:0006888 | endoplasmic reticulum to Golgi vesicle-mediated transport | 9.65107E-10 |
| BP | GO:0019221 | cytokine-mediated signaling pathway | 1.054E-09 |
| BP | GO:0002460 | adaptive immune response based on somatic recombination of immune receptors built from immunoglobulin superfamily domains | 3.41699E-09 |
| BP | GO:1901136 | carbohydrate derivative catabolic process | 4.18676E-09 |
| BP | GO:0002274 | myeloid leukocyte activation | 4.24926E-09 |
| BP | GO:0050863 | regulation of T cell activation | 6.43913E-09 |
| BP | GO:0043122 | regulation of I-kappaB kinase/NF-kappaB signaling | 6.76974E-09 |
| BP | GO:0007159 | leukocyte cell-cell adhesion | 7.1973E-09 |
| BP | GO:0018279 | protein N-linked glycosylation via asparagine | 7.82511E-09 |
| BP | GO:0002449 | lymphocyte mediated immunity | 8.72783E-09 |
| BP | GO:0006487 | protein N-linked glycosylation | 1.11609E-08 |
| BP | GO:0016485 | protein processing | 1.12672E-08 |
| BP | GO:0018196 | peptidyl-asparagine modification | 1.21992E-08 |
| BP | GO:0009101 | glycoprotein biosynthetic process | 1.38255E-08 |
| BP | GO:0045088 | regulation of innate immune response | 1.71608E-08 |
| BP | GO:0007249 | I-kappaB kinase/NF-kappaB signaling | 2.53155E-08 |
| BP | GO:0007596 | blood coagulation | 3.10447E-08 |
| BP | GO:0045785 | positive regulation of cell adhesion | 3.52872E-08 |
| BP | GO:0050817 | coagulation | 4.77965E-08 |
| BP | GO:0007599 | hemostasis | 5.20221E-08 |
| BP | GO:0002474 | antigen processing and presentation of peptide antigen via MHC class I | 8.20691E-08 |
| BP | GO:0016064 | immunoglobulin mediated immune response | 8.65821E-08 |
| BP | GO:0019724 | B cell mediated immunity | 1.1156E-07 |
| BP | GO:0022409 | positive regulation of cell-cell adhesion | 1.18037E-07 |
| BP | GO:1903131 | mononuclear cell differentiation | 1.6453E-07 |
| BP | GO:0046651 | lymphocyte proliferation | 1.66899E-07 |
| BP | GO:0052547 | regulation of peptidase activity | 1.87176E-07 |
| BP | GO:0070085 | glycosylation | 2.03637E-07 |
| BP | GO:0032943 | mononuclear cell proliferation | 2.18855E-07 |
| BP | GO:0032103 | positive regulation of response to external stimulus | 2.66841E-07 |
| BP | GO:0050670 | regulation of lymphocyte proliferation | 2.71502E-07 |
| BP | GO:0050777 | negative regulation of immune response | 2.75734E-07 |
| BP | GO:0030098 | lymphocyte differentiation | 2.84019E-07 |
| BP | GO:1903037 | regulation of leukocyte cell-cell adhesion | 3.12712E-07 |
| BP | GO:0002683 | negative regulation of immune system process | 3.2395E-07 |
| BP | GO:0052548 | regulation of endopeptidase activity | 3.41771E-07 |
| BP | GO:0032944 | regulation of mononuclear cell proliferation | 3.42473E-07 |
| BP | GO:0070661 | leukocyte proliferation | 4.38862E-07 |
| BP | GO:0019883 | antigen processing and presentation of endogenous antigen | 4.73472E-07 |
| BP | GO:1901658 | glycosyl compound catabolic process | 4.95268E-07 |
| BP | GO:0002697 | regulation of immune effector process | 5.35123E-07 |
| BP | GO:0022407 | regulation of cell-cell adhesion | 5.49373E-07 |
| BP | GO:0050870 | positive regulation of T cell activation | 6.21683E-07 |
| BP | GO:0002483 | antigen processing and presentation of endogenous peptide antigen | 6.23879E-07 |
| BP | GO:0006486 | protein glycosylation | 7.23985E-07 |
| BP | GO:0043413 | macromolecule glycosylation | 7.23985E-07 |
| BP | GO:0030217 | T cell differentiation | 8.0427E-07 |
| BP | GO:1903039 | positive regulation of leukocyte cell-cell adhesion | 8.32255E-07 |
| BP | GO:0050878 | regulation of body fluid levels | 8.8218E-07 |
| BP | GO:0050900 | leukocyte migration | 9.83552E-07 |
| BP | GO:0002381 | immunoglobulin production involved in immunoglobulin-mediated immune response | 1.16912E-06 |
| BP | GO:0046718 | viral entry into host cell | 1.20717E-06 |
| BP | GO:0031349 | positive regulation of defense response | 1.27856E-06 |
| BP | GO:0002833 | positive regulation of response to biotic stimulus | 1.30869E-06 |
| BP | GO:0001913 | T cell mediated cytotoxicity | 1.41997E-06 |
| BP | GO:0070663 | regulation of leukocyte proliferation | 1.5562E-06 |
| BP | GO:0034976 | response to endoplasmic reticulum stress | 1.90358E-06 |
| BP | GO:0042098 | T cell proliferation | 2.21081E-06 |
| BP | GO:0044409 | entry into host | 2.32347E-06 |
| BP | GO:0002224 | toll-like receptor signaling pathway | 2.84798E-06 |
| BP | GO:0034341 | response to interferon-gamma | 3.17199E-06 |
| BP | GO:0002366 | leukocyte activation involved in immune response | 3.32763E-06 |
| BP | GO:0019377 | glycolipid catabolic process | 3.64797E-06 |
| BP | GO:0002699 | positive regulation of immune effector process | 3.6683E-06 |
| BP | GO:0001819 | positive regulation of cytokine production | 3.76876E-06 |
| BP | GO:0002221 | pattern recognition receptor signaling pathway | 3.89606E-06 |
| BP | GO:0006457 | protein folding | 3.99528E-06 |
| BP | GO:0002263 | cell activation involved in immune response | 4.23337E-06 |
| BP | GO:0045861 | negative regulation of proteolysis | 4.338E-06 |
| BP | GO:0051346 | negative regulation of hydrolase activity | 4.39302E-06 |
| BP | GO:0016139 | glycoside catabolic process | 4.53084E-06 |
| BP | GO:0002440 | production of molecular mediator of immune response | 5.05258E-06 |
| BP | GO:0048193 | Golgi vesicle transport | 5.67987E-06 |
| BP | GO:0050766 | positive regulation of phagocytosis | 5.92832E-06 |
| BP | GO:0002696 | positive regulation of leukocyte activation | 6.33724E-06 |
| BP | GO:0002407 | dendritic cell chemotaxis | 6.82404E-06 |
| BP | GO:0032963 | collagen metabolic process | 7.15855E-06 |
| BP | GO:0034975 | protein folding in endoplasmic reticulum | 8.10794E-06 |
| BP | GO:0046598 | positive regulation of viral entry into host cell | 8.10794E-06 |
| BP | GO:0075294 | positive regulation by symbiont of entry into host | 8.10794E-06 |
| BP | GO:1902105 | regulation of leukocyte differentiation | 9.53573E-06 |
| BP | GO:0002444 | myeloid leukocyte mediated immunity | 9.91268E-06 |
| BP | GO:0045058 | T cell selection | 1.00345E-05 |
| BP | GO:0030199 | collagen fibril organization | 1.0874E-05 |
| BP | GO:0044403 | biological process involved in symbiotic interaction | 1.13208E-05 |
| BP | GO:0050867 | positive regulation of cell activation | 1.25301E-05 |
| BP | GO:0042129 | regulation of T cell proliferation | 1.32387E-05 |
| BP | GO:0002764 | immune response-regulating signaling pathway | 1.33183E-05 |
| BP | GO:0031638 | zymogen activation | 1.45432E-05 |
| BP | GO:0070665 | positive regulation of leukocyte proliferation | 1.50966E-05 |
| BP | GO:0050671 | positive regulation of lymphocyte proliferation | 1.53947E-05 |
| BP | GO:0001914 | regulation of T cell mediated cytotoxicity | 1.62897E-05 |
| BP | GO:0052126 | movement in host environment | 1.65726E-05 |
| BP | GO:0032946 | positive regulation of mononuclear cell proliferation | 1.82346E-05 |
| BP | GO:0001916 | positive regulation of T cell mediated cytotoxicity | 1.90932E-05 |
| BP | GO:0019058 | viral life cycle | 2.00089E-05 |
| BP | GO:0002456 | T cell mediated immunity | 2.22887E-05 |
| BP | GO:0050691 | regulation of defense response to virus by host | 2.37567E-05 |
| BP | GO:0001909 | leukocyte mediated cytotoxicity | 2.79471E-05 |
| BP | GO:0006022 | aminoglycan metabolic process | 2.79471E-05 |
| BP | GO:0001959 | regulation of cytokine-mediated signaling pathway | 2.79591E-05 |
| BP | GO:0010466 | negative regulation of peptidase activity | 2.84065E-05 |
| BP | GO:0046596 | regulation of viral entry into host cell | 2.84517E-05 |
| BP | GO:0001906 | cell killing | 2.94017E-05 |
| BP | GO:0050764 | regulation of phagocytosis | 2.96499E-05 |
| BP | GO:0006026 | aminoglycan catabolic process | 3.00168E-05 |
| BP | GO:0043903 | regulation of biological process involved in symbiotic interaction | 3.08933E-05 |
| BP | GO:0071260 | cellular response to mechanical stimulus | 3.25916E-05 |
| BP | GO:0046479 | glycosphingolipid catabolic process | 3.26776E-05 |
| BP | GO:2001233 | regulation of apoptotic signaling pathway | 3.31332E-05 |
| BP | GO:0051251 | positive regulation of lymphocyte activation | 3.46815E-05 |
| BP | GO:0001961 | positive regulation of cytokine-mediated signaling pathway | 3.57532E-05 |
| BP | GO:0006643 | membrane lipid metabolic process | 3.62019E-05 |
| BP | GO:0036336 | dendritic cell migration | 3.71514E-05 |
| BP | GO:0071346 | cellular response to interferon-gamma | 3.89561E-05 |
| BP | GO:0033003 | regulation of mast cell activation | 4.01906E-05 |
| BP | GO:0097529 | myeloid leukocyte migration | 4.23451E-05 |
| BP | GO:0006664 | glycolipid metabolic process | 4.41587E-05 |
| BP | GO:0051701 | biological process involved in interaction with host | 4.4172E-05 |
| BP | GO:0045824 | negative regulation of innate immune response | 4.72228E-05 |
| BP | GO:0010951 | negative regulation of endopeptidase activity | 4.76077E-05 |
| BP | GO:0034112 | positive regulation of homotypic cell-cell adhesion | 4.78477E-05 |
| BP | GO:1903509 | liposaccharide metabolic process | 4.86256E-05 |
| BP | GO:0006986 | response to unfolded protein | 5.41277E-05 |
| BP | GO:1903900 | regulation of viral life cycle | 5.41277E-05 |
| BP | GO:0036230 | granulocyte activation | 5.57188E-05 |
| BP | GO:1901657 | glycosyl compound metabolic process | 5.86164E-05 |
| BP | GO:0046631 | alpha-beta T cell activation | 5.88995E-05 |
| BP | GO:0071216 | cellular response to biotic stimulus | 6.2741E-05 |
| BP | GO:0050792 | regulation of viral process | 6.33323E-05 |
| BP | GO:0002703 | regulation of leukocyte mediated immunity | 6.36841E-05 |
| BP | GO:0033209 | tumor necrosis factor-mediated signaling pathway | 6.44512E-05 |
| BP | GO:0060759 | regulation of response to cytokine stimulus | 6.53647E-05 |
| BP | GO:0043368 | positive T cell selection | 6.73196E-05 |
| BP | GO:0046466 | membrane lipid catabolic process | 6.73196E-05 |
| BP | GO:0140448 | signaling receptor ligand precursor processing | 6.73196E-05 |
| BP | GO:0034109 | homotypic cell-cell adhesion | 7.94249E-05 |
| BP | GO:0052372 | modulation by symbiont of entry into host | 8.81355E-05 |
| BP | GO:0060760 | positive regulation of response to cytokine stimulus | 9.14155E-05 |
| BP | GO:1903706 | regulation of hemopoiesis | 9.17469E-05 |
| BP | GO:0019885 | antigen processing and presentation of endogenous peptide antigen via MHC class I | 9.39547E-05 |
| BP | GO:0016032 | viral process | 9.90513E-05 |
| BP | GO:0002698 | negative regulation of immune effector process | 0.000100737 |
| BP | GO:0018126 | protein hydroxylation | 0.000102709 |
| BP | GO:0030168 | platelet activation | 0.000106789 |
| BP | GO:0002832 | negative regulation of response to biotic stimulus | 0.000109791 |
| BP | GO:0006909 | phagocytosis | 0.00011415 |
| BP | GO:0002237 | response to molecule of bacterial origin | 0.000114945 |
| BP | GO:0006890 | retrograde vesicle-mediated transport, Golgi to endoplasmic reticulum | 0.000117412 |
| BP | GO:1905897 | regulation of response to endoplasmic reticulum stress | 0.000128775 |
| BP | GO:0071219 | cellular response to molecule of bacterial origin | 0.000133563 |
| BP | GO:0003158 | endothelium development | 0.00013469 |
| BP | GO:0002573 | myeloid leukocyte differentiation | 0.000142894 |
| BP | GO:0045446 | endothelial cell differentiation | 0.000153342 |
| BP | GO:0042119 | neutrophil activation | 0.000159918 |
| BP | GO:0070527 | platelet aggregation | 0.000165447 |
| BP | GO:0002695 | negative regulation of leukocyte activation | 0.00016925 |
| BP | GO:0032677 | regulation of interleukin-8 production | 0.000183594 |
| BP | GO:0051098 | regulation of binding | 0.000190255 |
| BP | GO:0030203 | glycosaminoglycan metabolic process | 0.000195012 |
| BP | GO:0031293 | membrane protein intracellular domain proteolysis | 0.000197361 |
| BP | GO:0032637 | interleukin-8 production | 0.000200266 |
| BP | GO:0042102 | positive regulation of T cell proliferation | 0.000200266 |
| BP | GO:0050866 | negative regulation of cell activation | 0.000200392 |
| BP | GO:0010803 | regulation of tumor necrosis factor-mediated signaling pathway | 0.000227062 |
| BP | GO:0002230 | positive regulation of defense response to virus by host | 0.000231489 |
| BP | GO:0002709 | regulation of T cell mediated immunity | 0.000232653 |
| BP | GO:0050727 | regulation of inflammatory response | 0.000233371 |
| BP | GO:0035966 | response to topologically incorrect protein | 0.000244216 |
| BP | GO:0001912 | positive regulation of leukocyte mediated cytotoxicity | 0.000256792 |
| BP | GO:0030198 | extracellular matrix organization | 0.000268085 |
| BP | GO:0009612 | response to mechanical stimulus | 0.000269084 |
| BP | GO:0032956 | regulation of actin cytoskeleton organization | 0.000270641 |
| BP | GO:0043062 | extracellular structure organization | 0.000280096 |
| BP | GO:0046514 | ceramide catabolic process | 0.000280624 |
| BP | GO:1903902 | positive regulation of viral life cycle | 0.000280624 |
| BP | GO:0050688 | regulation of defense response to virus | 0.000283841 |
| BP | GO:0016554 | cytidine to uridine editing | 0.000289258 |
| BP | GO:0045229 | external encapsulating structure organization | 0.000305542 |
| BP | GO:0007015 | actin filament organization | 0.000325899 |
| BP | GO:0002363 | alpha-beta T cell lineage commitment | 0.000354545 |
| BP | GO:0016137 | glycoside metabolic process | 0.000354545 |
| BP | GO:0070269 | pyroptosis | 0.000354545 |
| BP | GO:0002275 | myeloid cell activation involved in immune response | 0.000366003 |
| BP | GO:0034612 | response to tumor necrosis factor | 0.000374265 |
| BP | GO:0002700 | regulation of production of molecular mediator of immune response | 0.000379286 |
| BP | GO:0016486 | peptide hormone processing | 0.00039276 |
| BP | GO:0071356 | cellular response to tumor necrosis factor | 0.000397725 |
| BP | GO:0035437 | maintenance of protein localization in endoplasmic reticulum | 0.000408252 |
| BP | GO:1900426 | positive regulation of defense response to bacterium | 0.000408252 |
| BP | GO:0031663 | lipopolysaccharide-mediated signaling pathway | 0.000409285 |
| BP | GO:0097193 | intrinsic apoptotic signaling pathway | 0.000417391 |
| BP | GO:0031589 | cell-substrate adhesion | 0.000434575 |
| BP | GO:0034110 | regulation of homotypic cell-cell adhesion | 0.000462515 |
| BP | GO:0045920 | negative regulation of exocytosis | 0.000462515 |
| BP | GO:0097502 | mannosylation | 0.000462515 |
| BP | GO:0030225 | macrophage differentiation | 0.000500139 |
| BP | GO:0043300 | regulation of leukocyte degranulation | 0.000500139 |
| BP | GO:0016052 | carbohydrate catabolic process | 0.000501625 |
| BP | GO:0001885 | endothelial cell development | 0.000509333 |
| BP | GO:0032970 | regulation of actin filament-based process | 0.000536399 |
| BP | GO:0002755 | MyD88-dependent toll-like receptor signaling pathway | 0.000545447 |
| BP | GO:0006213 | pyrimidine nucleoside metabolic process | 0.000545447 |
| BP | GO:0046348 | amino sugar catabolic process | 0.000558482 |
| BP | GO:0002286 | T cell activation involved in immune response | 0.000563036 |
| BP | GO:0031343 | positive regulation of cell killing | 0.000566303 |
| BP | GO:0045576 | mast cell activation | 0.000566303 |
| BP | GO:0097530 | granulocyte migration | 0.000568169 |
| BP | GO:0032609 | interferon-gamma production | 0.000605551 |
| BP | GO:0032649 | regulation of interferon-gamma production | 0.000605551 |
| BP | GO:0071222 | cellular response to lipopolysaccharide | 0.000628181 |
| BP | GO:0002367 | cytokine production involved in immune response | 0.000654685 |
| BP | GO:0002685 | regulation of leukocyte migration | 0.000660228 |
| BP | GO:0060333 | interferon-gamma-mediated signaling pathway | 0.000665669 |
| BP | GO:0098581 | detection of external biotic stimulus | 0.000665669 |
| BP | GO:1903306 | negative regulation of regulated secretory pathway | 0.000665669 |
| BP | GO:0071674 | mononuclear cell migration | 0.000732615 |
| BP | GO:0002468 | dendritic cell antigen processing and presentation | 0.000744167 |
| BP | GO:0019511 | peptidyl-proline hydroxylation | 0.000744167 |
| BP | GO:0097191 | extrinsic apoptotic signaling pathway | 0.000764879 |
| BP | GO:0032496 | response to lipopolysaccharide | 0.000779335 |
| BP | GO:0001910 | regulation of leukocyte mediated cytotoxicity | 0.000798105 |
| BP | GO:0006027 | glycosaminoglycan catabolic process | 0.000804666 |
| BP | GO:0002285 | lymphocyte activation involved in immune response | 0.000811904 |
| BP | GO:1903076 | regulation of protein localization to plasma membrane | 0.000825915 |
| BP | GO:0045619 | regulation of lymphocyte differentiation | 0.000846794 |
| BP | GO:0006968 | cellular defense response | 0.000910165 |
| BP | GO:0006672 | ceramide metabolic process | 0.000959311 |
| BP | GO:0046634 | regulation of alpha-beta T cell activation | 0.000959311 |
| BP | GO:1903531 | negative regulation of secretion by cell | 0.000963891 |
| BP | GO:0110053 | regulation of actin filament organization | 0.000966582 |
| BP | GO:0046633 | alpha-beta T cell proliferation | 0.000967494 |
| BP | GO:0046135 | pyrimidine nucleoside catabolic process | 0.000969577 |
| BP | GO:1901163 | regulation of trophoblast cell migration | 0.000969577 |
| BP | GO:2001044 | regulation of integrin-mediated signaling pathway | 0.000969577 |
| BP | GO:0050868 | negative regulation of T cell activation | 0.001051887 |
| BP | GO:0050729 | positive regulation of inflammatory response | 0.001088229 |
| BP | GO:2000116 | regulation of cysteine-type endopeptidase activity | 0.001115433 |
| BP | GO:0002360 | T cell lineage commitment | 0.001146085 |
| BP | GO:0090025 | regulation of monocyte chemotaxis | 0.001146085 |
| BP | GO:2001234 | negative regulation of apoptotic signaling pathway | 0.001167562 |
| BP | GO:0051048 | negative regulation of secretion | 0.001208682 |
| BP | GO:0002819 | regulation of adaptive immune response | 0.00122234 |
| BP | GO:0016045 | detection of bacterium | 0.001238999 |
| BP | GO:0033631 | cell-cell adhesion mediated by integrin | 0.001238999 |
| BP | GO:0061450 | trophoblast cell migration | 0.001238999 |
| BP | GO:0090594 | inflammatory response to wounding | 0.001238999 |
| BP | GO:0030574 | collagen catabolic process | 0.001259066 |
| BP | GO:0034142 | toll-like receptor 4 signaling pathway | 0.001259066 |
| BP | GO:0034314 | Arp2/3 complex-mediated actin nucleation | 0.001259066 |
| BP | GO:0007566 | embryo implantation | 0.001260907 |
| BP | GO:0045071 | negative regulation of viral genome replication | 0.001260907 |
| BP | GO:1990266 | neutrophil migration | 0.001277317 |
| BP | GO:0048525 | negative regulation of viral process | 0.001303698 |
| BP | GO:0033619 | membrane protein proteolysis | 0.001398727 |
| BP | GO:0030595 | leukocyte chemotaxis | 0.001525545 |
| BP | GO:0045580 | regulation of T cell differentiation | 0.001543609 |
| BP | GO:0002711 | positive regulation of T cell mediated immunity | 0.001548028 |
| BP | GO:0002886 | regulation of myeloid leukocyte mediated immunity | 0.001548028 |
| BP | GO:0006488 | dolichol-linked oligosaccharide biosynthetic process | 0.001556715 |
| BP | GO:0022604 | regulation of cell morphogenesis | 0.001568323 |
| BP | GO:0034656 | nucleobase-containing small molecule catabolic process | 0.001584333 |
| BP | GO:0038094 | Fc-gamma receptor signaling pathway | 0.001584333 |
| BP | GO:1900101 | regulation of endoplasmic reticulum unfolded protein response | 0.001584333 |
| BP | GO:0043299 | leukocyte degranulation | 0.001602552 |
| BP | GO:0006509 | membrane protein ectodomain proteolysis | 0.00161342 |
| BP | GO:0016050 | vesicle organization | 0.001628266 |
| BP | GO:0072659 | protein localization to plasma membrane | 0.001639572 |
| BP | GO:0006687 | glycosphingolipid metabolic process | 0.001709451 |
| BP | GO:0051235 | maintenance of location | 0.001718729 |
| BP | GO:0032729 | positive regulation of interferon-gamma production | 0.001743873 |
| BP | GO:0070972 | protein localization to endoplasmic reticulum | 0.001743873 |
| BP | GO:0043200 | response to amino acid | 0.001796048 |
| BP | GO:0071496 | cellular response to external stimulus | 0.001819885 |
| BP | GO:0002377 | immunoglobulin production | 0.001830189 |
| BP | GO:0002706 | regulation of lymphocyte mediated immunity | 0.001842841 |
| BP | GO:0002862 | negative regulation of inflammatory response to antigenic stimulus | 0.001844496 |
| BP | GO:0030149 | sphingolipid catabolic process | 0.001844496 |
| BP | GO:1901264 | carbohydrate derivative transport | 0.001894858 |
| BP | GO:0006490 | oligosaccharide-lipid intermediate biosynthetic process | 0.001926971 |
| BP | GO:2001185 | regulation of CD8-positive, alpha-beta T cell activation | 0.001926971 |
| BP | GO:0032757 | positive regulation of interleukin-8 production | 0.002071298 |
| BP | GO:0043304 | regulation of mast cell degranulation | 0.002134591 |
| BP | GO:0002761 | regulation of myeloid leukocyte differentiation | 0.002179579 |
| BP | GO:0002718 | regulation of cytokine production involved in immune response | 0.002190488 |
| BP | GO:0042063 | gliogenesis | 0.002224505 |
| BP | GO:0031348 | negative regulation of defense response | 0.002243762 |
| BP | GO:0060326 | cell chemotaxis | 0.002260756 |
| BP | GO:0008064 | regulation of actin polymerization or depolymerization | 0.002262523 |
| BP | GO:0009311 | oligosaccharide metabolic process | 0.002273074 |
| BP | GO:0002577 | regulation of antigen processing and presentation | 0.002353961 |
| BP | GO:0006901 | vesicle coating | 0.002353961 |
| BP | GO:0016553 | base conversion or substitution editing | 0.002353961 |
| BP | GO:0036498 | IRE1-mediated unfolded protein response | 0.002353961 |
| BP | GO:0043373 | CD4-positive, alpha-beta T cell lineage commitment | 0.002353961 |
| BP | GO:0098543 | detection of other organism | 0.002353961 |
| BP | GO:1900424 | regulation of defense response to bacterium | 0.002353961 |
| BP | GO:0030832 | regulation of actin filament length | 0.002384534 |
| BP | GO:0002431 | Fc receptor mediated stimulatory signaling pathway | 0.002456571 |
| BP | GO:0030833 | regulation of actin filament polymerization | 0.002468039 |
| BP | GO:0006900 | vesicle budding from membrane | 0.002489674 |
| BP | GO:0009620 | response to fungus | 0.002489674 |
| BP | GO:1903018 | regulation of glycoprotein metabolic process | 0.002489674 |
| BP | GO:0043410 | positive regulation of MAPK cascade | 0.002502722 |
| BP | GO:0035646 | endosome to melanosome transport | 0.002509968 |
| BP | GO:0043485 | endosome to pigment granule transport | 0.002509968 |
| BP | GO:0048194 | Golgi vesicle budding | 0.002509968 |
| BP | GO:0048757 | pigment granule maturation | 0.002509968 |
| BP | GO:1901731 | positive regulation of platelet aggregation | 0.002509968 |
| BP | GO:0007030 | Golgi organization | 0.002511859 |
| BP | GO:0031341 | regulation of cell killing | 0.002516654 |
| BP | GO:0098869 | cellular oxidant detoxification | 0.002516654 |
| BP | GO:0030099 | myeloid cell differentiation | 0.002530159 |
| BP | GO:0009615 | response to virus | 0.00260998 |
| BP | GO:0045089 | positive regulation of innate immune response | 0.002610301 |
| BP | GO:1903305 | regulation of regulated secretory pathway | 0.002610301 |
| BP | GO:0006665 | sphingolipid metabolic process | 0.002644666 |
| BP | GO:0051250 | negative regulation of lymphocyte activation | 0.002644666 |
| BP | GO:0000041 | transition metal ion transport | 0.002693573 |
| BP | GO:0008630 | intrinsic apoptotic signaling pathway in response to DNA damage | 0.002693573 |
| BP | GO:0070613 | regulation of protein processing | 0.002721805 |
| BP | GO:0042509 | regulation of tyrosine phosphorylation of STAT protein | 0.002810986 |
| BP | GO:0033006 | regulation of mast cell activation involved in immune response | 0.002812401 |
| BP | GO:1901797 | negative regulation of signal transduction by p53 class mediator | 0.002812401 |
| BP | GO:1902106 | negative regulation of leukocyte differentiation | 0.002880192 |
| BP | GO:0032760 | positive regulation of tumor necrosis factor production | 0.003076878 |
| BP | GO:2001238 | positive regulation of extrinsic apoptotic signaling pathway | 0.00313753 |
| BP | GO:0002446 | neutrophil mediated immunity | 0.003204049 |
| BP | GO:0035633 | maintenance of blood-brain barrier | 0.003204049 |
| BP | GO:0044319 | wound healing, spreading of cells | 0.003204049 |
| BP | GO:0090505 | epiboly involved in wound healing | 0.003204049 |
| BP | GO:1905898 | positive regulation of response to endoplasmic reticulum stress | 0.003204049 |
| BP | GO:0002704 | negative regulation of leukocyte mediated immunity | 0.003235542 |
| BP | GO:0042531 | positive regulation of tyrosine phosphorylation of STAT protein | 0.003235542 |
| BP | GO:1903038 | negative regulation of leukocyte cell-cell adhesion | 0.003247037 |
| BP | GO:0008625 | extrinsic apoptotic signaling pathway via death domain receptors | 0.003261687 |
| BP | GO:2000117 | negative regulation of cysteine-type endopeptidase activity | 0.003261687 |
| BP | GO:1904375 | regulation of protein localization to cell periphery | 0.003333143 |
| BP | GO:0010038 | response to metal ion | 0.003376089 |
| BP | GO:0033860 | regulation of NAD(P)H oxidase activity | 0.003377196 |
| BP | GO:0043589 | skin morphogenesis | 0.003377196 |
| BP | GO:0090481 | pyrimidine nucleotide-sugar transmembrane transport | 0.003377196 |
| BP | GO:0043369 | CD4-positive or CD8-positive, alpha-beta T cell lineage commitment | 0.00339453 |
| BP | GO:1903034 | regulation of response to wounding | 0.00339708 |
| BP | GO:1902903 | regulation of supramolecular fiber organization | 0.00341653 |
| BP | GO:0008347 | glial cell migration | 0.00347037 |
| BP | GO:1903317 | regulation of protein maturation | 0.003518614 |
| BP | GO:0090504 | epiboly | 0.003633483 |
| BP | GO:1903707 | negative regulation of hemopoiesis | 0.00373108 |
| BP | GO:0007160 | cell-matrix adhesion | 0.00374582 |
| BP | GO:0007260 | tyrosine phosphorylation of STAT protein | 0.003766575 |
| BP | GO:0046635 | positive regulation of alpha-beta T cell activation | 0.003820146 |
| BP | GO:0030041 | actin filament polymerization | 0.00392579 |
| BP | GO:1903557 | positive regulation of tumor necrosis factor superfamily cytokine production | 0.003971804 |
| BP | GO:0048199 | vesicle targeting, to, from or within Golgi | 0.004016054 |
| BP | GO:1902254 | negative regulation of intrinsic apoptotic signaling pathway by p53 class mediator | 0.004016054 |
| BP | GO:0007029 | endoplasmic reticulum organization | 0.004040643 |
| BP | GO:1903510 | mucopolysaccharide metabolic process | 0.004040643 |
| BP | GO:0045730 | respiratory burst | 0.004102668 |
| BP | GO:0046640 | regulation of alpha-beta T cell proliferation | 0.004102668 |
| BP | GO:0072529 | pyrimidine-containing compound catabolic process | 0.004102668 |
| BP | GO:0061077 | chaperone-mediated protein folding | 0.004140893 |
| BP | GO:0050730 | regulation of peptidyl-tyrosine phosphorylation | 0.004159307 |
| BP | GO:0010718 | positive regulation of epithelial to mesenchymal transition | 0.004213854 |
| BP | GO:0033059 | cellular pigmentation | 0.004213854 |
| BP | GO:0051099 | positive regulation of binding | 0.004313618 |
| BP | GO:2001242 | regulation of intrinsic apoptotic signaling pathway | 0.004313618 |
| BP | GO:0002424 | T cell mediated immune response to tumor cell | 0.004406508 |
| BP | GO:0002887 | negative regulation of myeloid leukocyte mediated immunity | 0.004406508 |
| BP | GO:0006216 | cytidine catabolic process | 0.004406508 |
| BP | GO:0009972 | cytidine deamination | 0.004406508 |
| BP | GO:0015780 | nucleotide-sugar transmembrane transport | 0.004406508 |
| BP | GO:0032817 | regulation of natural killer cell proliferation | 0.004406508 |
| BP | GO:0034135 | regulation of toll-like receptor 2 signaling pathway | 0.004406508 |
| BP | GO:0038063 | collagen-activated tyrosine kinase receptor signaling pathway | 0.004406508 |
| BP | GO:0043476 | pigment accumulation | 0.004406508 |
| BP | GO:0043482 | cellular pigment accumulation | 0.004406508 |
| BP | GO:0046087 | cytidine metabolic process | 0.004406508 |
| BP | GO:0072182 | regulation of nephron tubule epithelial cell differentiation | 0.004406508 |
| BP | GO:1901142 | insulin metabolic process | 0.004406508 |
| BP | GO:1905245 | regulation of aspartic-type peptidase activity | 0.004406508 |
| BP | GO:0001101 | response to acid chemical | 0.004420546 |
| BP | GO:0071887 | leukocyte apoptotic process | 0.004489582 |
| BP | GO:0010810 | regulation of cell-substrate adhesion | 0.004561655 |
| BP | GO:0002253 | activation of immune response | 0.004612063 |
| BP | GO:0002701 | negative regulation of production of molecular mediator of immune response | 0.004613556 |
| BP | GO:0008360 | regulation of cell shape | 0.004656871 |
| BP | GO:0007035 | vacuolar acidification | 0.004710175 |
| BP | GO:0010560 | positive regulation of glycoprotein biosynthetic process | 0.004710175 |
| BP | GO:0045649 | regulation of macrophage differentiation | 0.004710175 |
| BP | GO:0050855 | regulation of B cell receptor signaling pathway | 0.004710175 |
| BP | GO:0002822 | regulation of adaptive immune response based on somatic recombination of immune receptors built from immunoglobulin superfamily domains | 0.00473157 |
| BP | GO:0007259 | receptor signaling pathway via JAK-STAT | 0.00473157 |
| BP | GO:0051650 | establishment of vesicle localization | 0.00473157 |
| BP | GO:0002824 | positive regulation of adaptive immune response based on somatic recombination of immune receptors built from immunoglobulin superfamily domains | 0.004767437 |
| BP | GO:0030330 | DNA damage response, signal transduction by p53 class mediator | 0.004843075 |
| BP | GO:1903036 | positive regulation of response to wounding | 0.004843075 |
| BP | GO:0051651 | maintenance of location in cell | 0.004942742 |
| BP | GO:0051607 | defense response to virus | 0.004995904 |
| BP | GO:0006790 | sulfur compound metabolic process | 0.005023372 |
| BP | GO:0046632 | alpha-beta T cell differentiation | 0.005058474 |
| BP | GO:0045010 | actin nucleation | 0.005068806 |
| BP | GO:1905517 | macrophage migration | 0.005068806 |
| BP | GO:2001236 | regulation of extrinsic apoptotic signaling pathway | 0.005138061 |
| BP | GO:0140546 | defense response to symbiont | 0.005167852 |
| BP | GO:0009595 | detection of biotic stimulus | 0.005168084 |
| BP | GO:0043687 | post-translational protein modification | 0.005168084 |
| BP | GO:0061041 | regulation of wound healing | 0.005477162 |
| BP | GO:0009164 | nucleoside catabolic process | 0.005480557 |
| BP | GO:0031639 | plasminogen activation | 0.005480557 |
| BP | GO:0032469 | endoplasmic reticulum calcium ion homeostasis | 0.005480557 |
| BP | GO:1903077 | negative regulation of protein localization to plasma membrane | 0.005480557 |
| BP | GO:0030166 | proteoglycan biosynthetic process | 0.005540989 |
| BP | GO:0060337 | type I interferon signaling pathway | 0.005540989 |
| BP | GO:0008154 | actin polymerization or depolymerization | 0.00554841 |
| BP | GO:0009313 | oligosaccharide catabolic process | 0.005605985 |
| BP | GO:0045006 | DNA deamination | 0.005605985 |
| BP | GO:0070486 | leukocyte aggregation | 0.005605985 |
| BP | GO:0072540 | T-helper 17 cell lineage commitment | 0.005605985 |
| BP | GO:1900103 | positive regulation of endoplasmic reticulum unfolded protein response | 0.005605985 |
| BP | GO:1903265 | positive regulation of tumor necrosis factor-mediated signaling pathway | 0.005605985 |
| BP | GO:0045669 | positive regulation of osteoblast differentiation | 0.005631307 |
| BP | GO:0048489 | synaptic vesicle transport | 0.005768168 |
| BP | GO:2001235 | positive regulation of apoptotic signaling pathway | 0.005769863 |
| BP | GO:0045862 | positive regulation of proteolysis | 0.005849257 |
| BP | GO:0002228 | natural killer cell mediated immunity | 0.006059631 |
| BP | GO:1902107 | positive regulation of leukocyte differentiation | 0.006217991 |
| BP | GO:1903708 | positive regulation of hemopoiesis | 0.006217991 |
| BP | GO:0031664 | regulation of lipopolysaccharide-mediated signaling pathway | 0.00633072 |
| BP | GO:0002821 | positive regulation of adaptive immune response | 0.006362702 |
| BP | GO:1990748 | cellular detoxification | 0.006362702 |
| BP | GO:0072595 | maintenance of protein localization in organelle | 0.0064157 |
| BP | GO:1905521 | regulation of macrophage migration | 0.0064157 |
| BP | GO:0002292 | T cell differentiation involved in immune response | 0.006511798 |
| BP | GO:0030968 | endoplasmic reticulum unfolded protein response | 0.006511798 |
| BP | GO:0034121 | regulation of toll-like receptor signaling pathway | 0.006511798 |
| BP | GO:0006816 | calcium ion transport | 0.006545718 |
| BP | GO:0010559 | regulation of glycoprotein biosynthetic process | 0.006580582 |
| BP | GO:0071357 | cellular response to type I interferon | 0.006580582 |
| BP | GO:0090303 | positive regulation of wound healing | 0.006580582 |
| BP | GO:1900024 | regulation of substrate adhesion-dependent cell spreading | 0.006580582 |
| BP | GO:0017157 | regulation of exocytosis | 0.006796291 |
| BP | GO:0043281 | regulation of cysteine-type endopeptidase activity involved in apoptotic process | 0.006796291 |
| BP | GO:0001787 | natural killer cell proliferation | 0.006982577 |
| BP | GO:0035563 | positive regulation of chromatin binding | 0.006982577 |
| BP | GO:0043301 | negative regulation of leukocyte degranulation | 0.006982577 |
| BP | GO:0045059 | positive thymic T cell selection | 0.006982577 |
| BP | GO:0046131 | pyrimidine ribonucleoside metabolic process | 0.006982577 |
| BP | GO:0046133 | pyrimidine ribonucleoside catabolic process | 0.006982577 |
| BP | GO:0072160 | nephron tubule epithelial cell differentiation | 0.006982577 |
| BP | GO:0098760 | response to interleukin-7 | 0.006982577 |
| BP | GO:0098761 | cellular response to interleukin-7 | 0.006982577 |
| BP | GO:1903894 | regulation of IRE1-mediated unfolded protein response | 0.006982577 |
| BP | GO:0032640 | tumor necrosis factor production | 0.007029468 |
| BP | GO:0032680 | regulation of tumor necrosis factor production | 0.007029468 |
| BP | GO:0002705 | positive regulation of leukocyte mediated immunity | 0.007064598 |
| BP | GO:0002708 | positive regulation of lymphocyte mediated immunity | 0.007104727 |
| BP | GO:0030501 | positive regulation of bone mineralization | 0.007112542 |
| BP | GO:1900026 | positive regulation of substrate adhesion-dependent cell spreading | 0.007112542 |
| BP | GO:0002262 | myeloid cell homeostasis | 0.007139013 |
| BP | GO:0002418 | immune response to tumor cell | 0.007264027 |
| BP | GO:0016540 | protein autoprocessing | 0.007264027 |
| BP | GO:0036037 | CD8-positive, alpha-beta T cell activation | 0.007264027 |
| BP | GO:1903020 | positive regulation of glycoprotein metabolic process | 0.007264027 |
| BP | GO:1904376 | negative regulation of protein localization to cell periphery | 0.007264027 |
| BP | GO:0032102 | negative regulation of response to external stimulus | 0.00746528 |
| BP | GO:0033077 | T cell differentiation in thymus | 0.007490789 |
| BP | GO:0097696 | receptor signaling pathway via STAT | 0.007643022 |
| BP | GO:0034620 | cellular response to unfolded protein | 0.007703389 |
| BP | GO:0006903 | vesicle targeting | 0.007860523 |
| BP | GO:0042771 | intrinsic apoptotic signaling pathway in response to DNA damage by p53 class mediator | 0.007860523 |
| BP | GO:0072332 | intrinsic apoptotic signaling pathway by p53 class mediator | 0.008019175 |
| BP | GO:0007162 | negative regulation of cell adhesion | 0.00812529 |
| BP | GO:0010955 | negative regulation of protein processing | 0.008283684 |
| BP | GO:0032438 | melanosome organization | 0.008283684 |
| BP | GO:0034123 | positive regulation of toll-like receptor signaling pathway | 0.008283684 |
| BP | GO:0090330 | regulation of platelet aggregation | 0.008283684 |
| BP | GO:1903318 | negative regulation of protein maturation | 0.008283684 |
| BP | GO:0048524 | positive regulation of viral process | 0.008395339 |
| BP | GO:0002836 | positive regulation of response to tumor cell | 0.008542179 |
| BP | GO:0002839 | positive regulation of immune response to tumor cell | 0.008542179 |
| BP | GO:0005980 | glycogen catabolic process | 0.008542179 |
| BP | GO:0010755 | regulation of plasminogen activation | 0.008542179 |
| BP | GO:0043518 | negative regulation of DNA damage response, signal transduction by p53 class mediator | 0.008542179 |
| BP | GO:1902166 | negative regulation of intrinsic apoptotic signaling pathway in response to DNA damage by p53 class mediator | 0.008542179 |
| BP | GO:0048872 | homeostasis of number of cells | 0.008565572 |
| BP | GO:0071706 | tumor necrosis factor superfamily cytokine production | 0.008641273 |
| BP | GO:1903555 | regulation of tumor necrosis factor superfamily cytokine production | 0.008641273 |
| BP | GO:0051452 | intracellular pH reduction | 0.008661434 |
| BP | GO:0051100 | negative regulation of binding | 0.008907081 |
| BP | GO:0051648 | vesicle localization | 0.009361375 |
| BP | GO:0030970 | retrograde protein transport, ER to cytosol | 0.009392727 |
| BP | GO:0048753 | pigment granule organization | 0.009392727 |
| BP | GO:0097066 | response to thyroid hormone | 0.009392727 |
| BP | GO:1903513 | endoplasmic reticulum to cytosol transport | 0.009392727 |
| BP | GO:0002861 | regulation of inflammatory response to antigenic stimulus | 0.009517024 |
| BP | GO:0032781 | positive regulation of ATP-dependent activity | 0.009517024 |
| BP | GO:0035710 | CD4-positive, alpha-beta T cell activation | 0.009715949 |
| BP | GO:2001243 | negative regulation of intrinsic apoptotic signaling pathway | 0.009715949 |
| BP | GO:0032355 | response to estradiol | 0.009731404 |
| BP | GO:0097237 | cellular response to toxic substance | 0.009731404 |
| BP | GO:0050731 | positive regulation of peptidyl-tyrosine phosphorylation | 0.009738496 |
| BP | GO:0043367 | CD4-positive, alpha-beta T cell differentiation | 0.009769228 |
| BP | GO:0070059 | intrinsic apoptotic signaling pathway in response to endoplasmic reticulum stress | 0.009788687 |
| BP | GO:1990778 | protein localization to cell periphery | 0.00997552 |
| BP | GO:0009251 | glucan catabolic process | 0.010289703 |
| BP | GO:0035751 | regulation of lysosomal lumen pH | 0.010289703 |
| BP | GO:0045651 | positive regulation of macrophage differentiation | 0.010289703 |
| BP | GO:0045869 | negative regulation of single stranded viral RNA replication via double stranded DNA intermediate | 0.010289703 |
| BP | GO:0048207 | vesicle targeting, rough ER to cis-Golgi | 0.010289703 |
| BP | GO:0048208 | COPII vesicle coating | 0.010289703 |
| BP | GO:1903975 | regulation of glial cell migration | 0.010289703 |
| BP | GO:2000696 | regulation of epithelial cell differentiation involved in kidney development | 0.010289703 |
| BP | GO:0009411 | response to UV | 0.010318516 |
| BP | GO:0010952 | positive regulation of peptidase activity | 0.010528037 |
| BP | GO:0009116 | nucleoside metabolic process | 0.010543757 |
| BP | GO:0032507 | maintenance of protein location in cell | 0.010543757 |
| BP | GO:0034340 | response to type I interferon | 0.010543757 |
| BP | GO:0031330 | negative regulation of cellular catabolic process | 0.010921806 |
| BP | GO:0072331 | signal transduction by p53 class mediator | 0.010996235 |
| BP | GO:0046467 | membrane lipid biosynthetic process | 0.011286306 |
| BP | GO:0006749 | glutathione metabolic process | 0.01133921 |
| BP | GO:0042093 | T-helper cell differentiation | 0.01133921 |
| BP | GO:0097028 | dendritic cell differentiation | 0.011399014 |
| BP | GO:0055072 | iron ion homeostasis | 0.011782793 |
| BP | GO:0001782 | B cell homeostasis | 0.011890243 |
| BP | GO:0006613 | cotranslational protein targeting to membrane | 0.011890243 |
| BP | GO:0007163 | establishment or maintenance of cell polarity | 0.011913562 |
| BP | GO:0032635 | interleukin-6 production | 0.011929617 |
| BP | GO:0032675 | regulation of interleukin-6 production | 0.011929617 |
| BP | GO:0042116 | macrophage activation | 0.012095548 |
| BP | GO:0050854 | regulation of antigen receptor-mediated signaling pathway | 0.012176113 |
| BP | GO:0002295 | T-helper cell lineage commitment | 0.012229139 |
| BP | GO:0006677 | glycosylceramide metabolic process | 0.012229139 |
| BP | GO:0034134 | toll-like receptor 2 signaling pathway | 0.012229139 |
| BP | GO:0038065 | collagen-activated signaling pathway | 0.012229139 |
| BP | GO:0044247 | cellular polysaccharide catabolic process | 0.012229139 |
| BP | GO:0097067 | cellular response to thyroid hormone stimulus | 0.012229139 |
| BP | GO:1902165 | regulation of intrinsic apoptotic signaling pathway in response to DNA damage by p53 class mediator | 0.012229139 |
| BP | GO:0043393 | regulation of protein binding | 0.012254855 |
| BP | GO:0098754 | detoxification | 0.012321321 |
| BP | GO:0010543 | regulation of platelet activation | 0.012428677 |
| BP | GO:0033628 | regulation of cell adhesion mediated by integrin | 0.012428677 |
| BP | GO:0061028 | establishment of endothelial barrier | 0.012428677 |
| BP | GO:0033627 | cell adhesion mediated by integrin | 0.012516083 |
| BP | GO:0007229 | integrin-mediated signaling pathway | 0.012752012 |
| BP | GO:0045621 | positive regulation of lymphocyte differentiation | 0.012752012 |
| BP | GO:0010950 | positive regulation of endopeptidase activity | 0.012922215 |
| BP | GO:0032612 | interleukin-1 production | 0.013031597 |
| BP | GO:0032652 | regulation of interleukin-1 production | 0.013031597 |
| BP | GO:0002294 | CD4-positive, alpha-beta T cell differentiation involved in immune response | 0.013055513 |
| BP | GO:0046513 | ceramide biosynthetic process | 0.013055513 |
| BP | GO:0072678 | T cell migration | 0.013055513 |
| BP | GO:0045069 | regulation of viral genome replication | 0.013281665 |
| BP | GO:0035456 | response to interferon-beta | 0.013283908 |
| BP | GO:0071353 | cellular response to interleukin-4 | 0.013283908 |
| BP | GO:0071711 | basement membrane organization | 0.013283908 |
| BP | GO:0090114 | COPII-coated vesicle budding | 0.013283908 |
| BP | GO:1902235 | regulation of endoplasmic reticulum stress-induced intrinsic apoptotic signaling pathway | 0.013283908 |
| BP | GO:1902253 | regulation of intrinsic apoptotic signaling pathway by p53 class mediator | 0.013283908 |
| BP | GO:0002762 | negative regulation of myeloid leukocyte differentiation | 0.013519539 |
| BP | GO:0022408 | negative regulation of cell-cell adhesion | 0.013686637 |
| BP | GO:0071214 | cellular response to abiotic stimulus | 0.013775668 |
| BP | GO:0104004 | cellular response to environmental stimulus | 0.013775668 |
| BP | GO:0002287 | alpha-beta T cell activation involved in immune response | 0.013978439 |
| BP | GO:0002293 | alpha-beta T cell differentiation involved in immune response | 0.013978439 |
| BP | GO:0034446 | substrate adhesion-dependent cell spreading | 0.014142698 |
| BP | GO:0070482 | response to oxygen levels | 0.014162331 |
| BP | GO:0018212 | peptidyl-tyrosine modification | 0.014240308 |
| BP | GO:0003159 | morphogenesis of an endothelium | 0.014363624 |
| BP | GO:0036035 | osteoclast development | 0.014363624 |
| BP | GO:0036499 | PERK-mediated unfolded protein response | 0.014363624 |
| BP | GO:0042339 | keratan sulfate metabolic process | 0.014363624 |
| BP | GO:0060263 | regulation of respiratory burst | 0.014363624 |
| BP | GO:0061154 | endothelial tube morphogenesis | 0.014363624 |
| BP | GO:0010959 | regulation of metal ion transport | 0.014498636 |
| BP | GO:0002715 | regulation of natural killer cell mediated immunity | 0.014673095 |
| BP | GO:0035088 | establishment or maintenance of apical/basal cell polarity | 0.014673095 |
| BP | GO:0043303 | mast cell degranulation | 0.014673095 |
| BP | GO:0060425 | lung morphogenesis | 0.014673095 |
| BP | GO:0061245 | establishment or maintenance of bipolar cell polarity | 0.014673095 |
| BP | GO:1905476 | negative regulation of protein localization to membrane | 0.014777334 |
| BP | GO:0051656 | establishment of organelle localization | 0.014856203 |
| BP | GO:1901222 | regulation of NIK/NF-kappaB signaling | 0.014878013 |
| BP | GO:0001776 | leukocyte homeostasis | 0.014912626 |
| BP | GO:0097581 | lamellipodium organization | 0.014912626 |
| BP | GO:0031532 | actin cytoskeleton reorganization | 0.015640695 |
| BP | GO:0006979 | response to oxidative stress | 0.015732079 |
| BP | GO:0002064 | epithelial cell development | 0.015789993 |
| BP | GO:0010823 | negative regulation of mitochondrion organization | 0.015890783 |
| BP | GO:0045778 | positive regulation of ossification | 0.015890783 |
| BP | GO:0002548 | monocyte chemotaxis | 0.015958864 |
| BP | GO:0019079 | viral genome replication | 0.016363436 |
| BP | GO:0002335 | mature B cell differentiation | 0.016372658 |
| BP | GO:0070670 | response to interleukin-4 | 0.016372658 |
| BP | GO:0000272 | polysaccharide catabolic process | 0.016695496 |
| BP | GO:0045091 | regulation of single stranded viral RNA replication via double stranded DNA intermediate | 0.016695496 |
| BP | GO:0050818 | regulation of coagulation | 0.017018303 |
| BP | GO:0006887 | exocytosis | 0.017085876 |
| BP | GO:0002279 | mast cell activation involved in immune response | 0.017173977 |
| BP | GO:0002448 | mast cell mediated immunity | 0.017173977 |
| BP | GO:0038093 | Fc receptor signaling pathway | 0.017173977 |
| BP | GO:0050819 | negative regulation of coagulation | 0.017173977 |
| BP | GO:0070169 | positive regulation of biomineral tissue development | 0.017173977 |
| BP | GO:0097479 | synaptic vesicle localization | 0.017173977 |
| BP | GO:0051051 | negative regulation of transport | 0.017261455 |
| BP | GO:0007044 | cell-substrate junction assembly | 0.017619237 |
| BP | GO:0051492 | regulation of stress fiber assembly | 0.017619237 |
| BP | GO:0072593 | reactive oxygen species metabolic process | 0.017827934 |
| BP | GO:0042267 | natural killer cell mediated cytotoxicity | 0.018125142 |
| BP | GO:0045766 | positive regulation of angiogenesis | 0.018182001 |
| BP | GO:1904018 | positive regulation of vasculature development | 0.018182001 |
| BP | GO:0072503 | cellular divalent inorganic cation homeostasis | 0.018339587 |
| BP | GO:0110151 | positive regulation of biomineralization | 0.018523989 |
| BP | GO:1902743 | regulation of lamellipodium organization | 0.018523989 |
| BP | GO:0030101 | natural killer cell activation | 0.018593585 |
| BP | GO:0045637 | regulation of myeloid cell differentiation | 0.018752612 |
| BP | GO:0055074 | calcium ion homeostasis | 0.018835937 |
| BP | GO:0002716 | negative regulation of natural killer cell mediated immunity | 0.019226355 |
| BP | GO:0002837 | regulation of immune response to tumor cell | 0.019226355 |
| BP | GO:0039692 | single stranded viral RNA replication via double stranded DNA intermediate | 0.019226355 |
| BP | GO:0042454 | ribonucleoside catabolic process | 0.019226355 |
| BP | GO:0042953 | lipoprotein transport | 0.019226355 |
| BP | GO:0090026 | positive regulation of monocyte chemotaxis | 0.019226355 |
| BP | GO:0032732 | positive regulation of interleukin-1 production | 0.019280284 |
| BP | GO:0043462 | regulation of ATP-dependent activity | 0.019280284 |
| BP | GO:0007565 | female pregnancy | 0.019540365 |
| BP | GO:0045055 | regulated exocytosis | 0.019604263 |
| BP | GO:0001818 | negative regulation of cytokine production | 0.019848588 |
| BP | GO:0050931 | pigment cell differentiation | 0.019876627 |
| BP | GO:0071675 | regulation of mononuclear cell migration | 0.019883071 |
| BP | GO:0070372 | regulation of ERK1 and ERK2 cascade | 0.02002653 |
| BP | GO:0055076 | transition metal ion homeostasis | 0.02028025 |
| BP | GO:0001666 | response to hypoxia | 0.020458168 |
| BP | GO:0007040 | lysosome organization | 0.020484606 |
| BP | GO:0080171 | lytic vacuole organization | 0.020484606 |
| BP | GO:0005996 | monosaccharide metabolic process | 0.020752265 |
| BP | GO:0035967 | cellular response to topologically incorrect protein | 0.020820931 |
| BP | GO:0007568 | aging | 0.020876305 |
| BP | GO:0061025 | membrane fusion | 0.020876305 |
| BP | GO:0050921 | positive regulation of chemotaxis | 0.021138292 |
| BP | GO:0002707 | negative regulation of lymphocyte mediated immunity | 0.021429395 |
| BP | GO:0002763 | positive regulation of myeloid leukocyte differentiation | 0.021429395 |
| BP | GO:0043470 | regulation of carbohydrate catabolic process | 0.021429395 |
| BP | GO:0001503 | ossification | 0.021578191 |
| BP | GO:0034329 | cell junction assembly | 0.021578191 |
| BP | GO:0002437 | inflammatory response to antigenic stimulus | 0.021738952 |
| BP | GO:0043536 | positive regulation of blood vessel endothelial cell migration | 0.021738952 |
| BP | GO:0070227 | lymphocyte apoptotic process | 0.021738952 |
| BP | GO:0071230 | cellular response to amino acid stimulus | 0.021738952 |
| BP | GO:0032091 | negative regulation of protein binding | 0.021742324 |
| BP | GO:0009119 | ribonucleoside metabolic process | 0.021788622 |
| BP | GO:0042092 | type 2 immune response | 0.021788622 |
| BP | GO:0045454 | cell redox homeostasis | 0.021788622 |
| BP | GO:0051084 | 'de novo' post-translational protein folding | 0.021788622 |
| BP | GO:0032069 | regulation of nuclease activity | 0.021957114 |
| BP | GO:0033630 | positive regulation of cell adhesion mediated by integrin | 0.021957114 |
| BP | GO:0034035 | purine ribonucleoside bisphosphate metabolic process | 0.021957114 |
| BP | GO:0044872 | lipoprotein localization | 0.021957114 |
| BP | GO:0050427 | 3'-phosphoadenosine 5'-phosphosulfate metabolic process | 0.021957114 |
| BP | GO:0150105 | protein localization to cell-cell junction | 0.021957114 |
| BP | GO:0002687 | positive regulation of leukocyte migration | 0.022022148 |
| BP | GO:0038061 | NIK/NF-kappaB signaling | 0.022022148 |
| BP | GO:0048284 | organelle fusion | 0.022022148 |
| BP | GO:0044706 | multi-multicellular organism process | 0.022112781 |
| BP | GO:0072676 | lymphocyte migration | 0.022790002 |
| BP | GO:0002532 | production of molecular mediator involved in inflammatory response | 0.022869244 |
| BP | GO:0032755 | positive regulation of interleukin-6 production | 0.022869244 |
| BP | GO:0030183 | B cell differentiation | 0.0229322 |
| BP | GO:0010332 | response to gamma radiation | 0.022987082 |
| BP | GO:0018208 | peptidyl-proline modification | 0.022987082 |
| BP | GO:0002369 | T cell cytokine production | 0.023809218 |
| BP | GO:0002724 | regulation of T cell cytokine production | 0.023809218 |
| BP | GO:0032717 | negative regulation of interleukin-8 production | 0.023809218 |
| BP | GO:0002702 | positive regulation of production of molecular mediator of immune response | 0.023822164 |
| BP | GO:0060349 | bone morphogenesis | 0.024035848 |
| BP | GO:0150115 | cell-substrate junction organization | 0.024035848 |
| BP | GO:0071248 | cellular response to metal ion | 0.02406601 |
| BP | GO:0001667 | ameboidal-type cell migration | 0.024239561 |
| BP | GO:0001649 | osteoblast differentiation | 0.024262998 |
| BP | GO:0043154 | negative regulation of cysteine-type endopeptidase activity involved in apoptotic process | 0.024400943 |
| BP | GO:0051453 | regulation of intracellular pH | 0.024400943 |
| BP | GO:0045765 | regulation of angiogenesis | 0.024416006 |
| BP | GO:0060350 | endochondral bone morphogenesis | 0.024616173 |
| BP | GO:0051101 | regulation of DNA binding | 0.024886698 |
| BP | GO:0002834 | regulation of response to tumor cell | 0.024888048 |
| BP | GO:0006907 | pinocytosis | 0.024888048 |
| BP | GO:0006925 | inflammatory cell apoptotic process | 0.024888048 |
| BP | GO:0045061 | thymic T cell selection | 0.024888048 |
| BP | GO:0050765 | negative regulation of phagocytosis | 0.024888048 |
| BP | GO:0140467 | integrated stress response signaling | 0.024888048 |
| BP | GO:0019318 | hexose metabolic process | 0.024990108 |
| BP | GO:0018108 | peptidyl-tyrosine phosphorylation | 0.025201844 |
| BP | GO:0043254 | regulation of protein-containing complex assembly | 0.025813683 |
| BP | GO:0010001 | glial cell differentiation | 0.025898835 |
| BP | GO:0014902 | myotube differentiation | 0.025984053 |
| BP | GO:0006874 | cellular calcium ion homeostasis | 0.026057293 |
| BP | GO:0002011 | morphogenesis of an epithelial sheet | 0.026317644 |
| BP | GO:0048660 | regulation of smooth muscle cell proliferation | 0.026964853 |
| BP | GO:0032418 | lysosome localization | 0.027272392 |
| BP | GO:1990849 | vacuolar localization | 0.027272392 |
| BP | GO:0070374 | positive regulation of ERK1 and ERK2 cascade | 0.027538659 |
| BP | GO:0051702 | biological process involved in interaction with symbiont | 0.027779515 |
| BP | GO:0110020 | regulation of actomyosin structure organization | 0.027779515 |
| BP | GO:2000377 | regulation of reactive oxygen species metabolic process | 0.027888442 |
| BP | GO:0009895 | negative regulation of catabolic process | 0.027935789 |
| BP | GO:0045048 | protein insertion into ER membrane | 0.028018847 |
| BP | GO:0046641 | positive regulation of alpha-beta T cell proliferation | 0.028018847 |
| BP | GO:1900120 | regulation of receptor binding | 0.028018847 |
| BP | GO:0006826 | iron ion transport | 0.028092398 |
| BP | GO:0044275 | cellular carbohydrate catabolic process | 0.028180908 |
| BP | GO:1901342 | regulation of vasculature development | 0.028183756 |
| BP | GO:0001894 | tissue homeostasis | 0.028253585 |
| BP | GO:0002688 | regulation of leukocyte chemotaxis | 0.028278985 |
| BP | GO:0006029 | proteoglycan metabolic process | 0.028788435 |
| BP | GO:0009636 | response to toxic substance | 0.028868808 |
| BP | GO:1905475 | regulation of protein localization to membrane | 0.028915721 |
| BP | GO:0036293 | response to decreased oxygen levels | 0.028923468 |
| BP | GO:0010717 | regulation of epithelial to mesenchymal transition | 0.029110515 |
| BP | GO:0070997 | neuron death | 0.029531491 |
| BP | GO:0002429 | immune response-activating cell surface receptor signaling pathway | 0.029667873 |
| BP | GO:0002757 | immune response-activating signal transduction | 0.029667873 |
| BP | GO:0048659 | smooth muscle cell proliferation | 0.029927828 |
| BP | GO:0030500 | regulation of bone mineralization | 0.0303589 |
| BP | GO:0006040 | amino sugar metabolic process | 0.030533903 |
| BP | GO:0006458 | 'de novo' protein folding | 0.030533903 |
| BP | GO:0002768 | immune response-regulating cell surface receptor signaling pathway | 0.030783318 |
| BP | GO:0010634 | positive regulation of epithelial cell migration | 0.030964756 |
| BP | GO:0048771 | tissue remodeling | 0.030964756 |
| BP | GO:0003081 | regulation of systemic arterial blood pressure by renin-angiotensin | 0.031348657 |
| BP | GO:0017121 | plasma membrane phospholipid scrambling | 0.031348657 |
| BP | GO:0032740 | positive regulation of interleukin-17 production | 0.031348657 |
| BP | GO:0035561 | regulation of chromatin binding | 0.031348657 |
| BP | GO:0042730 | fibrinolysis | 0.031348657 |
| BP | GO:0072202 | cell differentiation involved in metanephros development | 0.031348657 |
| BP | GO:0030865 | cortical cytoskeleton organization | 0.031865009 |
| BP | GO:0032233 | positive regulation of actin filament bundle assembly | 0.031865009 |
| BP | GO:0034113 | heterotypic cell-cell adhesion | 0.031865009 |
| BP | GO:0050920 | regulation of chemotaxis | 0.031966493 |
| BP | GO:0050852 | T cell receptor signaling pathway | 0.031978217 |
| BP | GO:0051258 | protein polymerization | 0.032254824 |
| BP | GO:0018242 | protein O-linked glycosylation via serine | 0.032464393 |
| BP | GO:0018401 | peptidyl-proline hydroxylation to 4-hydroxy-L-proline | 0.032464393 |
| BP | GO:0032060 | bleb assembly | 0.032464393 |
| BP | GO:0032070 | regulation of deoxyribonuclease activity | 0.032464393 |
| BP | GO:0032819 | positive regulation of natural killer cell proliferation | 0.032464393 |
| BP | GO:0034356 | NAD biosynthesis via nicotinamide riboside salvage pathway | 0.032464393 |
| BP | GO:0044351 | macropinocytosis | 0.032464393 |
| BP | GO:0045657 | positive regulation of monocyte differentiation | 0.032464393 |
| BP | GO:0060368 | regulation of Fc receptor mediated stimulatory signaling pathway | 0.032464393 |
| BP | GO:0060397 | growth hormone receptor signaling pathway via JAK-STAT | 0.032464393 |
| BP | GO:0090557 | establishment of endothelial intestinal barrier | 0.032464393 |
| BP | GO:0098974 | postsynaptic actin cytoskeleton organization | 0.032464393 |
| BP | GO:1902884 | positive regulation of response to oxidative stress | 0.032464393 |
| BP | GO:1902946 | protein localization to early endosome | 0.032464393 |
| BP | GO:1902959 | regulation of aspartic-type endopeptidase activity involved in amyloid precursor protein catabolic process | 0.032464393 |
| BP | GO:0002347 | response to tumor cell | 0.03299931 |
| BP | GO:0031670 | cellular response to nutrient | 0.03299931 |
| BP | GO:0060338 | regulation of type I interferon-mediated signaling pathway | 0.03299931 |
| BP | GO:0070266 | necroptotic process | 0.03299931 |
| BP | GO:0010631 | epithelial cell migration | 0.033110211 |
| BP | GO:1902905 | positive regulation of supramolecular fiber organization | 0.033114155 |
| BP | GO:0031098 | stress-activated protein kinase signaling cascade | 0.033169354 |
| BP | GO:0032271 | regulation of protein polymerization | 0.033234285 |
| BP | GO:0042542 | response to hydrogen peroxide | 0.033281377 |
| BP | GO:0071621 | granulocyte chemotaxis | 0.033281377 |
| BP | GO:0030641 | regulation of cellular pH | 0.033665514 |
| BP | GO:0032731 | positive regulation of interleukin-1 beta production | 0.033864316 |
| BP | GO:0060393 | regulation of pathway-restricted SMAD protein phosphorylation | 0.033864316 |
| BP | GO:0043542 | endothelial cell migration | 0.033965452 |
| BP | GO:0032231 | regulation of actin filament bundle assembly | 0.034863528 |
| BP | GO:0062207 | regulation of pattern recognition receptor signaling pathway | 0.034863528 |
| BP | GO:0001911 | negative regulation of leukocyte mediated cytotoxicity | 0.034876126 |
| BP | GO:0006614 | SRP-dependent cotranslational protein targeting to membrane | 0.034876126 |
| BP | GO:0031579 | membrane raft organization | 0.034876126 |
| BP | GO:1901071 | glucosamine-containing compound metabolic process | 0.034876126 |
| BP | GO:1905523 | positive regulation of macrophage migration | 0.034876126 |
| BP | GO:0050672 | negative regulation of lymphocyte proliferation | 0.03540278 |
| BP | GO:0090132 | epithelium migration | 0.035405394 |
| BP | GO:2000146 | negative regulation of cell motility | 0.035405394 |
| BP | GO:0042554 | superoxide anion generation | 0.035577647 |
| BP | GO:0051893 | regulation of focal adhesion assembly | 0.035939801 |
| BP | GO:0090109 | regulation of cell-substrate junction assembly | 0.035939801 |
| BP | GO:2000242 | negative regulation of reproductive process | 0.035939801 |
| BP | GO:0070371 | ERK1 and ERK2 cascade | 0.036273716 |
| BP | GO:0030004 | cellular monovalent inorganic cation homeostasis | 0.036411334 |
| BP | GO:0030038 | contractile actin filament bundle assembly | 0.036411334 |
| BP | GO:0043149 | stress fiber assembly | 0.036411334 |
| BP | GO:0032945 | negative regulation of mononuclear cell proliferation | 0.037196703 |
| BP | GO:0071229 | cellular response to acid chemical | 0.037196703 |
| BP | GO:0010595 | positive regulation of endothelial cell migration | 0.037406286 |
| BP | GO:0044703 | multi-organism reproductive process | 0.037515457 |
| BP | GO:0062208 | positive regulation of pattern recognition receptor signaling pathway | 0.038269268 |
| BP | GO:1903573 | negative regulation of response to endoplasmic reticulum stress | 0.038269268 |
| BP | GO:0001783 | B cell apoptotic process | 0.038599439 |
| BP | GO:0002433 | immune response-regulating cell surface receptor signaling pathway involved in phagocytosis | 0.038599439 |
| BP | GO:0006517 | protein deglycosylation | 0.038599439 |
| BP | GO:0009435 | NAD biosynthetic process | 0.038599439 |
| BP | GO:0010954 | positive regulation of protein processing | 0.038599439 |
| BP | GO:0034143 | regulation of toll-like receptor 4 signaling pathway | 0.038599439 |
| BP | GO:0038096 | Fc-gamma receptor signaling pathway involved in phagocytosis | 0.038599439 |
| BP | GO:0060706 | cell differentiation involved in embryonic placenta development | 0.038599439 |
| BP | GO:0070102 | interleukin-6-mediated signaling pathway | 0.038599439 |
| BP | GO:0006612 | protein targeting to membrane | 0.038854213 |
| BP | GO:0001765 | membrane raft assembly | 0.038928214 |
| BP | GO:0001886 | endothelial cell morphogenesis | 0.038928214 |
| BP | GO:0002604 | regulation of dendritic cell antigen processing and presentation | 0.038928214 |
| BP | GO:0007042 | lysosomal lumen acidification | 0.038928214 |
| BP | GO:0032429 | regulation of phospholipase A2 activity | 0.038928214 |
| BP | GO:0033210 | leptin-mediated signaling pathway | 0.038928214 |
| BP | GO:0034145 | positive regulation of toll-like receptor 4 signaling pathway | 0.038928214 |
| BP | GO:0042118 | endothelial cell activation | 0.038928214 |
| BP | GO:0043471 | regulation of cellular carbohydrate catabolic process | 0.038928214 |
| BP | GO:0048251 | elastic fiber assembly | 0.038928214 |
| BP | GO:0061299 | retina vasculature morphogenesis in camera-type eye | 0.038928214 |
| BP | GO:0070944 | neutrophil-mediated killing of bacterium | 0.038928214 |
| BP | GO:0071803 | positive regulation of podosome assembly | 0.038928214 |
| BP | GO:1902065 | response to L-glutamate | 0.038928214 |
| BP | GO:1903897 | regulation of PERK-mediated unfolded protein response | 0.038928214 |
| BP | GO:1903977 | positive regulation of glial cell migration | 0.038928214 |
| BP | GO:1905668 | positive regulation of protein localization to endosome | 0.038928214 |
| BP | GO:2000392 | regulation of lamellipodium morphogenesis | 0.038928214 |
| BP | GO:2000508 | regulation of dendritic cell chemotaxis | 0.038928214 |
| BP | GO:2000644 | regulation of receptor catabolic process | 0.038928214 |
| BP | GO:2001223 | negative regulation of neuron migration | 0.038928214 |
| BP | GO:2001241 | positive regulation of extrinsic apoptotic signaling pathway in absence of ligand | 0.038928214 |
| BP | GO:0002312 | B cell activation involved in immune response | 0.039047749 |
| BP | GO:0019915 | lipid storage | 0.039047749 |
| BP | GO:0090130 | tissue migration | 0.039486458 |
| BP | GO:0031960 | response to corticosteroid | 0.039967896 |
| BP | GO:0055067 | monovalent inorganic cation homeostasis | 0.039967896 |
| BP | GO:0050728 | negative regulation of inflammatory response | 0.04018516 |
| BP | GO:0002260 | lymphocyte homeostasis | 0.040321408 |
| BP | GO:0006984 | ER-nucleus signaling pathway | 0.041074365 |
| BP | GO:0045047 | protein targeting to ER | 0.041074365 |
| BP | GO:0045197 | establishment or maintenance of epithelial cell apical/basal polarity | 0.041074365 |
| BP | GO:1902622 | regulation of neutrophil migration | 0.041074365 |
| BP | GO:2000273 | positive regulation of signaling receptor activity | 0.041074365 |
| BP | GO:0032611 | interleukin-1 beta production | 0.04132428 |
| BP | GO:0032651 | regulation of interleukin-1 beta production | 0.04132428 |
| BP | GO:0040013 | negative regulation of locomotion | 0.041581793 |
| BP | GO:0070588 | calcium ion transmembrane transport | 0.041623037 |
| BP | GO:0070304 | positive regulation of stress-activated protein kinase signaling cascade | 0.041861428 |
| BP | GO:0051271 | negative regulation of cellular component movement | 0.042093024 |
| BP | GO:0097305 | response to alcohol | 0.042312436 |
| BP | GO:0002719 | negative regulation of cytokine production involved in immune response | 0.042516366 |
| BP | GO:0010528 | regulation of transposition | 0.042516366 |
| BP | GO:0010529 | negative regulation of transposition | 0.042516366 |
| BP | GO:0034505 | tooth mineralization | 0.042516366 |
| BP | GO:0035459 | vesicle cargo loading | 0.042516366 |
| BP | GO:1900017 | positive regulation of cytokine production involved in inflammatory response | 0.042516366 |
| BP | GO:1903055 | positive regulation of extracellular matrix organization | 0.042516366 |
| BP | GO:2000108 | positive regulation of leukocyte apoptotic process | 0.042516366 |
| BP | GO:0060389 | pathway-restricted SMAD protein phosphorylation | 0.0426284 |
| BP | GO:0071677 | positive regulation of mononuclear cell migration | 0.0426284 |
| BP | GO:0001938 | positive regulation of endothelial cell proliferation | 0.043052982 |
| BP | GO:0001974 | blood vessel remodeling | 0.043992971 |
| BP | GO:0035987 | endodermal cell differentiation | 0.043992971 |
| BP | GO:0042269 | regulation of natural killer cell mediated cytotoxicity | 0.043992971 |
| BP | GO:0042398 | cellular modified amino acid biosynthetic process | 0.043992971 |
| BP | GO:0045581 | negative regulation of T cell differentiation | 0.043992971 |
| BP | GO:0085029 | extracellular matrix assembly | 0.043992971 |
| BP | GO:0050679 | positive regulation of epithelial cell proliferation | 0.044638039 |
| BP | GO:0006493 | protein O-linked glycosylation | 0.044947729 |
| BP | GO:1901224 | positive regulation of NIK/NF-kappaB signaling | 0.04501331 |
| BP | GO:0045667 | regulation of osteoblast differentiation | 0.045019071 |
| BP | GO:0002638 | negative regulation of immunoglobulin production | 0.045832556 |
| BP | GO:0003337 | mesenchymal to epithelial transition involved in metanephros morphogenesis | 0.045832556 |
| BP | GO:0006983 | ER overload response | 0.045832556 |
| BP | GO:0010635 | regulation of mitochondrial fusion | 0.045832556 |
| BP | GO:0010992 | ubiquitin recycling | 0.045832556 |
| BP | GO:0032490 | detection of molecule of bacterial origin | 0.045832556 |
| BP | GO:0034154 | toll-like receptor 7 signaling pathway | 0.045832556 |
| BP | GO:0034333 | adherens junction assembly | 0.045832556 |
| BP | GO:0043380 | regulation of memory T cell differentiation | 0.045832556 |
| BP | GO:0051608 | histamine transport | 0.045832556 |
| BP | GO:0060019 | radial glial cell differentiation | 0.045832556 |
| BP | GO:0070103 | regulation of interleukin-6-mediated signaling pathway | 0.045832556 |
| BP | GO:0070254 | mucus secretion | 0.045832556 |
| BP | GO:0070943 | neutrophil-mediated killing of symbiont cell | 0.045832556 |
| BP | GO:0072497 | mesenchymal stem cell differentiation | 0.045832556 |
| BP | GO:0097201 | negative regulation of transcription from RNA polymerase II promoter in response to stress | 0.045832556 |
| BP | GO:0099188 | postsynaptic cytoskeleton organization | 0.045832556 |
| BP | GO:1900227 | positive regulation of NLRP3 inflammasome complex assembly | 0.045832556 |
| BP | GO:1904953 | Wnt signaling pathway involved in midbrain dopaminergic neuron differentiation | 0.045832556 |
| BP | GO:1905522 | negative regulation of macrophage migration | 0.045832556 |
| BP | GO:1905666 | regulation of protein localization to endosome | 0.045832556 |
| BP | GO:0030336 | negative regulation of cell migration | 0.046289892 |
| BP | GO:0006829 | zinc ion transport | 0.046624288 |
| BP | GO:0008053 | mitochondrial fusion | 0.046624288 |
| BP | GO:0010592 | positive regulation of lamellipodium assembly | 0.046624288 |
| BP | GO:0019359 | nicotinamide nucleotide biosynthetic process | 0.046624288 |
| BP | GO:0019363 | pyridine nucleotide biosynthetic process | 0.046624288 |
| BP | GO:0031342 | negative regulation of cell killing | 0.046624288 |
| BP | GO:0033688 | regulation of osteoblast proliferation | 0.046624288 |
| BP | GO:0042983 | amyloid precursor protein biosynthetic process | 0.046624288 |
| BP | GO:0042984 | regulation of amyloid precursor protein biosynthetic process | 0.046624288 |
| BP | GO:0060544 | regulation of necroptotic process | 0.046624288 |
| BP | GO:0072539 | T-helper 17 cell differentiation | 0.046624288 |
| BP | GO:0080111 | DNA demethylation | 0.046624288 |
| BP | GO:1903319 | positive regulation of protein maturation | 0.046624288 |
| BP | GO:0044344 | cellular response to fibroblast growth factor stimulus | 0.046648916 |
| BP | GO:0032964 | collagen biosynthetic process | 0.047024967 |
| BP | GO:0043618 | regulation of transcription from RNA polymerase II promoter in response to stress | 0.047024967 |
| BP | GO:0048066 | developmental pigmentation | 0.047024967 |
| BP | GO:0002720 | positive regulation of cytokine production involved in immune response | 0.047476395 |
| BP | GO:0150116 | regulation of cell-substrate junction organization | 0.047476395 |
| BP | GO:0007266 | Rho protein signal transduction | 0.048329278 |
| BP | GO:0070664 | negative regulation of leukocyte proliferation | 0.049173514 |
| CC | GO:0005788 | endoplasmic reticulum lumen | 1.26431E-20 |
| CC | GO:0030134 | COPII-coated ER to Golgi transport vesicle | 1.53211E-17 |
| CC | GO:0034774 | secretory granule lumen | 6.52145E-17 |
| CC | GO:0005793 | endoplasmic reticulum-Golgi intermediate compartment | 9.00964E-17 |
| CC | GO:0060205 | cytoplasmic vesicle lumen | 9.219E-17 |
| CC | GO:0031983 | vesicle lumen | 1.15849E-16 |
| CC | GO:0005766 | primary lysosome | 1.30581E-16 |
| CC | GO:0042582 | azurophil granule | 1.30581E-16 |
| CC | GO:0042470 | melanosome | 6.98454E-16 |
| CC | GO:0048770 | pigment granule | 6.98454E-16 |
| CC | GO:0042611 | MHC protein complex | 9.03671E-16 |
| CC | GO:0005925 | focal adhesion | 2.24219E-15 |
| CC | GO:0030667 | secretory granule membrane | 3.34716E-15 |
| CC | GO:0030055 | cell-substrate junction | 5.06447E-15 |
| CC | GO:0030176 | integral component of endoplasmic reticulum membrane | 3.85582E-14 |
| CC | GO:0031227 | intrinsic component of endoplasmic reticulum membrane | 1.32777E-13 |
| CC | GO:0031301 | integral component of organelle membrane | 1.45499E-13 |
| CC | GO:0140534 | endoplasmic reticulum protein-containing complex | 1.80365E-13 |
| CC | GO:0098576 | lumenal side of membrane | 3.59487E-13 |
| CC | GO:0071556 | integral component of lumenal side of endoplasmic reticulum membrane | 4.22724E-13 |
| CC | GO:0098553 | lumenal side of endoplasmic reticulum membrane | 4.22724E-13 |
| CC | GO:0005775 | vacuolar lumen | 1.19063E-12 |
| CC | GO:0031300 | intrinsic component of organelle membrane | 1.84372E-12 |
| CC | GO:0005774 | vacuolar membrane | 2.08022E-12 |
| CC | GO:0005765 | lysosomal membrane | 3.95068E-12 |
| CC | GO:0098852 | lytic vacuole membrane | 3.95068E-12 |
| CC | GO:0042613 | MHC class II protein complex | 6.61319E-12 |
| CC | GO:0035578 | azurophil granule lumen | 1.28408E-11 |
| CC | GO:0030135 | coated vesicle | 2.51746E-11 |
| CC | GO:0062023 | collagen-containing extracellular matrix | 2.66865E-11 |
| CC | GO:0033116 | endoplasmic reticulum-Golgi intermediate compartment membrane | 6.03855E-11 |
| CC | GO:0043202 | lysosomal lumen | 2.71058E-09 |
| CC | GO:0098791 | Golgi apparatus subcompartment | 2.96283E-09 |
| CC | GO:0042581 | specific granule | 8.71617E-09 |
| CC | GO:0012507 | ER to Golgi transport vesicle membrane | 2.14206E-08 |
| CC | GO:0008250 | oligosaccharyltransferase complex | 4.94403E-08 |
| CC | GO:0030139 | endocytic vesicle | 8.9664E-08 |
| CC | GO:0045335 | phagocytic vesicle | 1.10844E-07 |
| CC | GO:0101002 | ficolin-1-rich granule | 1.13216E-07 |
| CC | GO:1904090 | peptidase inhibitor complex | 2.43388E-07 |
| CC | GO:0070820 | tertiary granule | 3.29496E-07 |
| CC | GO:0035577 | azurophil granule membrane | 7.68858E-07 |
| CC | GO:0005770 | late endosome | 9.34972E-07 |
| CC | GO:0030662 | coated vesicle membrane | 9.84339E-07 |
| CC | GO:0030666 | endocytic vesicle membrane | 1.06825E-06 |
| CC | GO:0045121 | membrane raft | 1.21816E-06 |
| CC | GO:0098857 | membrane microdomain | 1.2922E-06 |
| CC | GO:0030173 | integral component of Golgi membrane | 1.54458E-06 |
| CC | GO:0009897 | external side of plasma membrane | 1.62783E-06 |
| CC | GO:1904813 | ficolin-1-rich granule lumen | 3.30382E-06 |
| CC | GO:0031228 | intrinsic component of Golgi membrane | 3.42339E-06 |
| CC | GO:0030133 | transport vesicle | 7.71199E-06 |
| CC | GO:0034663 | endoplasmic reticulum chaperone complex | 8.24898E-06 |
| CC | GO:0005802 | trans-Golgi network | 8.35634E-06 |
| CC | GO:0031902 | late endosome membrane | 9.68079E-06 |
| CC | GO:0031985 | Golgi cisterna | 1.0671E-05 |
| CC | GO:0035579 | specific granule membrane | 1.29593E-05 |
| CC | GO:0030670 | phagocytic vesicle membrane | 1.36842E-05 |
| CC | GO:0030137 | COPI-coated vesicle | 2.98867E-05 |
| CC | GO:0098644 | complex of collagen trimers | 2.98867E-05 |
| CC | GO:0005795 | Golgi stack | 4.23435E-05 |
| CC | GO:0035580 | specific granule lumen | 7.28916E-05 |
| CC | GO:0030027 | lamellipodium | 9.30969E-05 |
| CC | GO:0031252 | cell leading edge | 9.76128E-05 |
| CC | GO:0030663 | COPI-coated vesicle membrane | 0.000129122 |
| CC | GO:0098636 | protein complex involved in cell adhesion | 0.000222833 |
| CC | GO:0070821 | tertiary granule membrane | 0.00026272 |
| CC | GO:0000138 | Golgi trans cisterna | 0.000293156 |
| CC | GO:0005583 | fibrillar collagen trimer | 0.000293156 |
| CC | GO:0005797 | Golgi medial cisterna | 0.000293156 |
| CC | GO:0098643 | banded collagen fibril | 0.000293156 |
| CC | GO:0015629 | actin cytoskeleton | 0.000358695 |
| CC | GO:0072562 | blood microparticle | 0.000376443 |
| CC | GO:0005911 | cell-cell junction | 0.000730811 |
| CC | GO:0030660 | Golgi-associated vesicle membrane | 0.000738071 |
| CC | GO:0005798 | Golgi-associated vesicle | 0.000751051 |
| CC | GO:0005581 | collagen trimer | 0.000891296 |
| CC | GO:1904724 | tertiary granule lumen | 0.001038539 |
| CC | GO:0030669 | clathrin-coated endocytic vesicle membrane | 0.001151279 |
| CC | GO:0031091 | platelet alpha granule | 0.001336597 |
| CC | GO:0031901 | early endosome membrane | 0.001630847 |
| CC | GO:0032580 | Golgi cisterna membrane | 0.001680027 |
| CC | GO:0005604 | basement membrane | 0.001809035 |
| CC | GO:0055037 | recycling endosome | 0.001875729 |
| CC | GO:0055038 | recycling endosome membrane | 0.001945846 |
| CC | GO:0005769 | early endosome | 0.002488234 |
| CC | GO:0032588 | trans-Golgi network membrane | 0.002577874 |
| CC | GO:0030658 | transport vesicle membrane | 0.002835517 |
| CC | GO:0031904 | endosome lumen | 0.003252725 |
| CC | GO:0019774 | proteasome core complex, beta-subunit complex | 0.003410482 |
| CC | GO:0071682 | endocytic vesicle lumen | 0.004066373 |
| CC | GO:0043296 | apical junction complex | 0.00411238 |
| CC | GO:0030136 | clathrin-coated vesicle | 0.004243162 |
| CC | GO:0005902 | microvillus | 0.004733975 |
| CC | GO:0045334 | clathrin-coated endocytic vesicle | 0.005061434 |
| CC | GO:0035861 | site of double-strand break | 0.00532653 |
| CC | GO:0005884 | actin filament | 0.005812623 |
| CC | GO:0001726 | ruffle | 0.005825 |
| CC | GO:0031528 | microvillus membrane | 0.006408437 |
| CC | GO:0043020 | NADPH oxidase complex | 0.007049745 |
| CC | GO:0090734 | site of DNA damage | 0.007403858 |
| CC | GO:0005791 | rough endoplasmic reticulum | 0.008168856 |
| CC | GO:1905369 | endopeptidase complex | 0.008168856 |
| CC | GO:0101003 | ficolin-1-rich granule membrane | 0.00853454 |
| CC | GO:0005811 | lipid droplet | 0.008843386 |
| CC | GO:0005771 | multivesicular body | 0.009949224 |
| CC | GO:0005790 | smooth endoplasmic reticulum | 0.010720531 |
| CC | GO:0019897 | extrinsic component of plasma membrane | 0.01127265 |
| CC | GO:0008305 | integrin complex | 0.012031249 |
| CC | GO:0005912 | adherens junction | 0.012227124 |
| CC | GO:0045178 | basal part of cell | 0.012776639 |
| CC | GO:0031093 | platelet alpha granule lumen | 0.013264947 |
| CC | GO:0033162 | melanosome membrane | 0.014497318 |
| CC | GO:0045009 | chitosome | 0.014497318 |
| CC | GO:0090741 | pigment granule membrane | 0.014497318 |
| CC | GO:0070971 | endoplasmic reticulum exit site | 0.014950137 |
| CC | GO:0016323 | basolateral plasma membrane | 0.015554568 |
| CC | GO:0030665 | clathrin-coated vesicle membrane | 0.015944568 |
| CC | GO:0016529 | sarcoplasmic reticulum | 0.017285261 |
| CC | GO:0044291 | cell-cell contact zone | 0.017285261 |
| CC | GO:0005839 | proteasome core complex | 0.019402352 |
| CC | GO:0097038 | perinuclear endoplasmic reticulum | 0.022156429 |
| CC | GO:0016460 | myosin II complex | 0.025112072 |
| CC | GO:0031258 | lamellipodium membrane | 0.025112072 |
| CC | GO:0016528 | sarcoplasm | 0.026199154 |
| CC | GO:0098562 | cytoplasmic side of membrane | 0.026320723 |
| CC | GO:0005657 | replication fork | 0.030331372 |
| CC | GO:0097197 | tetraspanin-enriched microdomain | 0.032670708 |
| CC | GO:0031256 | leading edge membrane | 0.032695226 |
| CC | GO:0009925 | basal plasma membrane | 0.033075093 |
| CC | GO:0030175 | filopodium | 0.033904054 |
| CC | GO:0001772 | immunological synapse | 0.038682746 |
| CC | GO:0002178 | palmitoyltransferase complex | 0.039172981 |
| CC | GO:0042589 | zymogen granule membrane | 0.039172981 |
| CC | GO:0099091 | postsynaptic specialization, intracellular component | 0.039172981 |
| CC | GO:1905286 | serine-type peptidase complex | 0.039172981 |
| CC | GO:0005635 | nuclear envelope | 0.042228836 |
| CC | GO:0032585 | multivesicular body membrane | 0.042883077 |
| CC | GO:1905368 | peptidase complex | 0.043732539 |
| CC | GO:0005776 | autophagosome | 0.045531443 |
| CC | GO:0036019 | endolysosome | 0.047022967 |
| CC | GO:0043596 | nuclear replication fork | 0.047022967 |
| MF | GO:0023026 | MHC class II protein complex binding | 3.60923E-09 |
| MF | GO:0016758 | hexosyltransferase activity | 4.64131E-08 |
| MF | GO:0023023 | MHC protein complex binding | 8.47604E-08 |
| MF | GO:0140375 | immune receptor activity | 1.41308E-07 |
| MF | GO:0016757 | glycosyltransferase activity | 3.02409E-07 |
| MF | GO:0016798 | hydrolase activity, acting on glycosyl bonds | 4.50277E-07 |
| MF | GO:0004553 | hydrolase activity, hydrolyzing O-glycosyl compounds | 1.02151E-06 |
| MF | GO:0032395 | MHC class II receptor activity | 5.93216E-06 |
| MF | GO:0005518 | collagen binding | 6.48054E-06 |
| MF | GO:0019864 | IgG binding | 1.06009E-05 |
| MF | GO:0030246 | carbohydrate binding | 1.07131E-05 |
| MF | GO:0003756 | protein disulfide isomerase activity | 1.10242E-05 |
| MF | GO:0016864 | intramolecular oxidoreductase activity, transposing S-S bonds | 1.10242E-05 |
| MF | GO:0004175 | endopeptidase activity | 1.15407E-05 |
| MF | GO:0042605 | peptide antigen binding | 1.31733E-05 |
| MF | GO:0004859 | phospholipase inhibitor activity | 2.80591E-05 |
| MF | GO:0045296 | cadherin binding | 3.86318E-05 |
| MF | GO:0019956 | chemokine binding | 5.27001E-05 |
| MF | GO:0016667 | oxidoreductase activity, acting on a sulfur group of donors | 5.48005E-05 |
| MF | GO:0097153 | cysteine-type endopeptidase activity involved in apoptotic process | 6.22128E-05 |
| MF | GO:0000030 | mannosyltransferase activity | 6.83128E-05 |
| MF | GO:0015035 | protein-disulfide reductase activity | 0.000114195 |
| MF | GO:0055102 | lipase inhibitor activity | 0.000121825 |
| MF | GO:0042608 | T cell receptor binding | 0.000159058 |
| MF | GO:0048407 | platelet-derived growth factor binding | 0.000243891 |
| MF | GO:0015036 | disulfide oxidoreductase activity | 0.000262073 |
| MF | GO:0061134 | peptidase regulator activity | 0.000276203 |
| MF | GO:0002020 | protease binding | 0.00028762 |
| MF | GO:0061135 | endopeptidase regulator activity | 0.000332026 |
| MF | GO:0004866 | endopeptidase inhibitor activity | 0.000452218 |
| MF | GO:0003779 | actin binding | 0.000518871 |
| MF | GO:0030414 | peptidase inhibitor activity | 0.000674458 |
| MF | GO:0044548 | S100 protein binding | 0.000687415 |
| MF | GO:0019865 | immunoglobulin binding | 0.00070049 |
| MF | GO:0016860 | intramolecular oxidoreductase activity | 0.000785304 |
| MF | GO:0004601 | peroxidase activity | 0.000997783 |
| MF | GO:0004857 | enzyme inhibitor activity | 0.001032411 |
| MF | GO:0005178 | integrin binding | 0.001062807 |
| MF | GO:0004197 | cysteine-type endopeptidase activity | 0.001170914 |
| MF | GO:0016209 | antioxidant activity | 0.0011744 |
| MF | GO:0004190 | aspartic-type endopeptidase activity | 0.001233233 |
| MF | GO:0005200 | structural constituent of cytoskeleton | 0.001347498 |
| MF | GO:0019955 | cytokine binding | 0.001363618 |
| MF | GO:0070001 | aspartic-type peptidase activity | 0.00146388 |
| MF | GO:0016684 | oxidoreductase activity, acting on peroxide as acceptor | 0.001556728 |
| MF | GO:0046527 | glucosyltransferase activity | 0.001906057 |
| MF | GO:0098641 | cadherin binding involved in cell-cell adhesion | 0.001906057 |
| MF | GO:0005126 | cytokine receptor binding | 0.001931597 |
| MF | GO:0042277 | peptide binding | 0.00235708 |
| MF | GO:0008236 | serine-type peptidase activity | 0.002361595 |
| MF | GO:0017171 | serine hydrolase activity | 0.002854717 |
| MF | GO:0005522 | profilin binding | 0.002936505 |
| MF | GO:0015165 | pyrimidine nucleotide-sugar transmembrane transporter activity | 0.002936505 |
| MF | GO:0016175 | superoxide-generating NAD(P)H oxidase activity | 0.002936505 |
| MF | GO:0047844 | deoxycytidine deaminase activity | 0.002936505 |
| MF | GO:0070008 | serine-type exopeptidase activity | 0.002936505 |
| MF | GO:0097199 | cysteine-type endopeptidase activity involved in apoptotic signaling pathway | 0.002936505 |
| MF | GO:0015026 | coreceptor activity | 0.003000308 |
| MF | GO:0005123 | death receptor binding | 0.003465955 |
| MF | GO:0019838 | growth factor binding | 0.003751066 |
| MF | GO:0070851 | growth factor receptor binding | 0.003751066 |
| MF | GO:0070513 | death domain binding | 0.003946236 |
| MF | GO:0097493 | structural molecule activity conferring elasticity | 0.003946236 |
| MF | GO:0004602 | glutathione peroxidase activity | 0.004134707 |
| MF | GO:0043394 | proteoglycan binding | 0.004591344 |
| MF | GO:0016493 | C-C chemokine receptor activity | 0.004885427 |
| MF | GO:0004126 | cytidine deaminase activity | 0.005142654 |
| MF | GO:0005338 | nucleotide-sugar transmembrane transporter activity | 0.005142654 |
| MF | GO:0035325 | Toll-like receptor binding | 0.005142654 |
| MF | GO:0046790 | virion binding | 0.005142654 |
| MF | GO:0097677 | STAT family protein binding | 0.005142654 |
| MF | GO:0048306 | calcium-dependent protein binding | 0.005230864 |
| MF | GO:0051087 | chaperone binding | 0.005328246 |
| MF | GO:1901505 | carbohydrate derivative transmembrane transporter activity | 0.005497773 |
| MF | GO:0019957 | C-C chemokine binding | 0.005722414 |
| MF | GO:0048029 | monosaccharide binding | 0.006025432 |
| MF | GO:0005544 | calcium-dependent phospholipid binding | 0.006028365 |
| MF | GO:0051015 | actin filament binding | 0.006482011 |
| MF | GO:0030247 | polysaccharide binding | 0.006649784 |
| MF | GO:0033218 | amide binding | 0.006661917 |
| MF | GO:0004869 | cysteine-type endopeptidase inhibitor activity | 0.00719992 |
| MF | GO:0001637 | G protein-coupled chemoattractant receptor activity | 0.007671457 |
| MF | GO:0004950 | chemokine receptor activity | 0.007671457 |
| MF | GO:0030020 | extracellular matrix structural constituent conferring tensile strength | 0.008053339 |
| MF | GO:0004298 | threonine-type endopeptidase activity | 0.00812916 |
| MF | GO:0017128 | phospholipid scramblase activity | 0.00812916 |
| MF | GO:0001618 | virus receptor activity | 0.008695044 |
| MF | GO:0043236 | laminin binding | 0.008791149 |
| MF | GO:0140272 | exogenous protein binding | 0.009318936 |
| MF | GO:0044183 | protein folding chaperone | 0.009840958 |
| MF | GO:0050664 | oxidoreductase activity, acting on NAD(P)H, oxygen as acceptor | 0.00993276 |
| MF | GO:0004896 | cytokine receptor activity | 0.0099341 |
| MF | GO:0004180 | carboxypeptidase activity | 0.0108294 |
| MF | GO:0001846 | opsonin binding | 0.011950256 |
| MF | GO:0050700 | CARD domain binding | 0.011950256 |
| MF | GO:0043621 | protein self-association | 0.012600145 |
| MF | GO:0008238 | exopeptidase activity | 0.013215512 |
| MF | GO:0098631 | cell adhesion mediator activity | 0.013553962 |
| MF | GO:0016722 | oxidoreductase activity, acting on metal ions | 0.014185518 |
| MF | GO:0005154 | epidermal growth factor receptor binding | 0.015974675 |
| MF | GO:0032813 | tumor necrosis factor receptor superfamily binding | 0.016793358 |
| MF | GO:0005201 | extracellular matrix structural constituent | 0.017301161 |
| MF | GO:0019239 | deaminase activity | 0.017748194 |
| MF | GO:0004252 | serine-type endopeptidase activity | 0.018688409 |
| MF | GO:0043022 | ribosome binding | 0.02035226 |
| MF | GO:0016853 | isomerase activity | 0.021425324 |
| MF | GO:0008234 | cysteine-type peptidase activity | 0.021708134 |
| MF | GO:0001848 | complement binding | 0.022221952 |
| MF | GO:0031418 | L-ascorbic acid binding | 0.022221952 |
| MF | GO:0043539 | protein serine/threonine kinase activator activity | 0.022890779 |
| MF | GO:1990782 | protein tyrosine kinase binding | 0.023114516 |
| MF | GO:0004867 | serine-type endopeptidase inhibitor activity | 0.031157579 |
| MF | GO:0051082 | unfolded protein binding | 0.03299011 |
| MF | GO:0003925 | G protein activity | 0.033551956 |
| MF | GO:0008194 | UDP-glycosyltransferase activity | 0.033648635 |
| MF | GO:0019203 | carbohydrate phosphatase activity | 0.035936571 |
| MF | GO:0043295 | glutathione binding | 0.035936571 |
| MF | GO:0050308 | sugar-phosphatase activity | 0.035936571 |
| MF | GO:0070003 | threonine-type peptidase activity | 0.036061608 |
| MF | GO:0044389 | ubiquitin-like protein ligase binding | 0.036174247 |
| MF | GO:0017124 | SH3 domain binding | 0.038867872 |
